# Recombinant laccase rPOXA 1B real-time, accelerated and molecular dynamics stability study

**DOI:** 10.1186/s12896-021-00698-3

**Published:** 2021-06-04

**Authors:** Leidy D. Ardila-Leal, Pedro A. Monterey-Gutiérrez, Raúl A. Poutou-Piñales, Balkys E. Quevedo-Hidalgo, Johan F. Galindo, Aura M. Pedroza-Rodríguez

**Affiliations:** 1 Departamento de Microbiología. Facultad de Ciencias. Pontificia Universidad Javeriana (PUJ). Bogotá, Laboratorio de Biotecnología Molecular, Grupo de Biotecnología Ambiental e Industrial (GBAI), Bogotá, D.C Colombia; 2 Vicerrectoría Académica. Universidad Antonio Nariño, Programa de Maestría y Doctorado en Educación Matemática, Bogotá, D.C Colombia; 3 Departamento de Microbiología. Facultad de Ciencias. Pontificia Universidad Javeriana (PUJ), Laboratorio de Biotecnología Aplicada, Grupo de Biotecnología Ambiental e Industrial (GBAI), Bogotá, D.C Colombia; 4 Departamento de Química, Universidad Nacional de Colombia, Bogotá, D.C Colombia; 5 Departamento de Microbiología. Facultad de Ciencias. Pontificia Universidad Javeriana (PUJ). Bogotá, Laboratorio de Microbiología Ambiental y de Suelos, Grupo de Biotecnología Ambiental e Industrial (GBAI), Bogotá, D.C Colombia

**Keywords:** rPOXA 1B, Storage conditions, Laccase, Real-time stability, Accelerated stability, Molecular dynamics

## Abstract

**Background:**

Laccases (EC 1.10.3.2) are multi-copper oxidoreductases with great biotechnological importance due to their high oxidative potential and utility for removing synthetic dyes, oxidizing phenolic compounds, and degrading pesticides, among others.

**Methods:**

A real-time stability study (RTS) was conducted for a year, by using enzyme concentrates from 3 batches (L1, L3, and L4). For which, five temperatures 243.15, 277.15, 298.15, 303.15, 308.15, and 313.15 K were assayed. Using RTS data and the Arrhenius equation, we calculated the rPOXA 1B accelerated stability (AS). Molecular dynamics (MD) computational study results were very close to those obtained experimentally at four different temperatures 241, 278, 298, and 314 K.

**Results:**

In the RTS, 101.16, 115.81, 75.23, 46.09, 5.81, and 4.83% of the relative enzyme activity were recovered, at respective assayed temperatures. AS study, showed that rPOXA 1B is stable at 240.98 ± 5.38, 277.40 ± 1.32 or 297.53 ± 3.88 K; with t_1/2_ values of 230.8, 46.2, and 12.6 months, respectively. Kinetic and thermodynamic parameters supported the high stability of rPOXA 1B, with an *E*_*d*_ value of 41.40 KJ mol^− 1^, a low variation of *K*_*M*_ and *V*_*max*_, at 240.98 ± 5.38, and 297.53 ± 3.88 K, and ∆G values showing deactivation reaction does not occur. The MD indicates that fluctuations in loop, coils or loops with hydrophilic or intermediate polarity amino acids as well as in some residues of POXA 1B 3D structure, increases with temperature; changing from three fluctuating residues at 278 K to six residues at 298 K, and nine residues at 314 K.

**Conclusions:**

Laccase rPOXA 1B demonstrated experimentally and computationally to be a stable enzyme, with t_1/2_ of 230.8, 46.2 or 12.6 months, if it is preserved impure without preservatives at temperatures of 240.98 ± 5.38, 277.40 ± 1.32 or 297.53 ± 3.88 K respectively; this study could be of great utility for large scale producers.

**Supplementary Information:**

The online version contains supplementary material available at 10.1186/s12896-021-00698-3.

## Background

Laccases (EC 1.10.3.2) are blue multi-copper oxidases (MCOs), usually monomeric and distributed in plants, insects, bacteria and fungi [1]. However, laccases from fungi have higher redox potentials (*E*_*o*_) [2, 3], fostering oxidation of chemicals as mono, di-, poly- and methoxy phenols, aromatic and aliphatic amines, hydroxy indoles, benzenethiols and inorganic/organic metal compounds [4–6]. Due to laccases low specificity [7], they are attractive for different biotechnological applications, including juice clarification [8], biosensor manufacturing (for agri-environmental and biomedical applications) [9], sawdust biotransformation [10], coloured wastewater treatment [11–13], detoxification of cellulose pulp black liquor [14], simultaneous bioconversion of plastics and waste [15], biodeterioration of low-density plastics [16], and oxidation of micropollutants [17].

The laccases catalytic centre contains four copper atoms distributed over CuT1, CuT2 and CuT3 sites, located between cupredoxin-like domains 1 and 3 [1, 18]. Site CuT1 is mononuclear and coordinated with D3 residues. CuT1 is considered as the indicator of the redox potential of the enzyme [7, 19] because it captures electrons from the substrate and transferring them to the trinuclear copper centre (TNC) of the enzyme [20]. The TNC is composed of one copper from the CuT2 site (mononuclear) and two coppers from the CuT3 site (binuclear - CuT3α and CuT3β) [7]. Copper ions coordinate with the D1 and D3 domains located residues, where the reduction of O_2_ to H_2_O occurs [1, 19, 21].

Due to water production during catalysis, it has become a promising “green tool” for industrial and environmental applications. Therefore, its production and commercial availability must increase [3].

Knowing the thermal stability and storage conditions of any enzyme is of great importance to determine potential industrial applications [22, 23]. However, each enzyme has self-structure and stability, and it depends for the most part on its chemical environment as pH, ionic force, among others, temperature [24, 25]. Reasons for which, is remarkable to determine the most favourable storage conditions, as a function of the enzyme’s shelf life [24], and to elucidate the necessity of incorporating preservatives agents to the final formulation.

For the study of the environment that favour enzyme and protein stability during storage, different conditions should be assayed (pH, temperature, NaCl concentration, presence of additives, preservatives and enzyme concentration, among others), [23]. Evaluating storage conditions at different temperatures is crucial since an increase in temperature augments the kinetic energy, leading to possible loss of enzyme activity and irreversible enzyme denaturation [26]. Additionally, it could also result in dynamic changes in its 3D structure. These changes could be more or less aggressive, depending on the enzyme’s thermostability [27].

Biological products are largely unstable; thus, it is customarily to store them under cold conditions. However, these storage conditions are not always the best as they involve high costs due to the maintenance of the cold chain [28]. Besides, enzyme denaturation can also occur under cold conditions [29]. Therefore, expiration dates are suggested based on collected real-time stability data. Accelerated stability studies are a complementary tool applied to proteins or enzymes with different applications [24].

Protein accelerated stability studies are performed usually at high temperatures (40 °C = 313.15 K), [24] as a tool to assess stability under adverse conditions [30]. Then employing Arrhenius formulas [31], the activation energy (*E*_*a*_) and half-life (t_1/2_) can be estimated; it is frequently for estimating protein and enzyme stability for pharmaceutical applications [24].

Accurate predictions of the thermal stability of enzymes and proteins are complex [32]. However, the combination of experimental results and computational analyses are effective for protein stability studies, as they allow rationalisation and interpretation of the experimental results [33]. Molecular dynamics (MD) simulations [34] explain the relationship between dynamics and the molecular mechanisms that stabilise the molecule under study through tools that measure flexibility and correlate it with function [33–35] as flexibility is a key property in protein activity and stability [36].

Some laccase MD analysis has focused on evaluating the influence of environmental conditions or the effect of the degree of glycosylation on structure, function and enzyme dynamics [18, 25]. Moreover, modified laccases by directed mutagenesis in the laboratory have been evaluating by MD [25, 37, 38].

The objectives of this work were, *i*) to propose the design of a more statistically based stability study, which would allow estimating at each sampling time with the same precision and confidence level the average enzyme activity (UL^− 1^) of rPOXA 1B laccase and thus support the stability study of other enzymes and proteins of non-pharmaceutical use [39], *ii)* use this statistical foundation to determine the real-time and accelerated stability of the recombinant rPOXA 1B enzyme from *P. ostreatus* produced in *P. pastoris* and *iii*) relate the real-time-stability with the in silico behaviour of the enzyme during molecular dynamics simulations.

## Materials and methods

### Pilot study of enzyme stability

To estimate rPOXA1B enzyme activity (UL^− 1^) variability at different temperatures a pilot study was performed. Fifteen vials were prepared (2 mL polypropylene screw-cap Microtube BIOLOGIX) containing 1 mL rPOXA 1B concentrate with an enzyme activity of 5877.31 ± 278.11 UL^− 1^ (~ 164.62 Umg^− 1^) placed at − 30, 4, 30, 40 and 50 °C, equivalent to absolute theoretical temperatures of 243.15, 277.15, 303.15, 313.15 and 323.15 K, respectively. Temperatures were selected based on a previous thermal stability study performed for 1 h of enzyme exposure [40]. Enzyme activity (UL^− 1^), [41], was measured at days 0, 15 and 30 (each one considered a sampling time), for each temperature. Sampling was performed at random, using a generator of random numbers with Stata V.14. Pilot study results were used to define the vial population size (***N***) and from this one, for each sampling time a sample size (*n*) was calculated. Estimated variances in the pilot study were used to appraise the variability of the process. Moreover, assuming homogeneity of variance, its combined variance was calculated with a similar statistical analysis as is used to estimate variance for a *t*-test for equal variances.

### rPOXA 1B concentrate batches

We use *P. pastoris X33/pGAPZαA-LaccPost-Stop Clone 1* [42] to produce rPOXA 1B batches on a 10 L bioreactor scale; following the production and concentration methodology previously described [40]. For the study, we used three random selected batches (L1, L3 and L4), wich enzyme activity per concentrate batch (preservative-free), were L1: 27,222.2 UL^− 1^; L3: 37,222.2 UL^− 1^ and L4: 34,236.1 UL^− 1^. Every batch was diluted with distilled water to match the initial enzyme activity of 16,575.50 ± 268.92 UL^− 1^ (758.71 ± 180.18 Umg^− 1^), time zero of the study without sampling.

### rPOXA 1B stability study: design and conditions

Based on the pilot stability study results, five temperatures were selected [243.15 K (stored in the freezer), 277.15 K (stored in the refrigerator), 298.15, 303.15, 308.15 and 313.15 K (stored in independent incubators)]. A thermo-hygrometer placed in each incubation equipment allows daily monitoring of temperature and relative humidity (RH) expressed in %, except for − 30 °C (243.15 K) and 4 °C (277.15 K) at which humidity is normally high. Temperature and RH values obtained throughout the study (1 year) were transformed to K and averaged at the end of the stability study to calculate both standard deviation (SD) during the length of the stability study. The stability study has a monthly periodicity (30 calendar days) until the first 6 months and later, every 2 months (60 days) to complete a year. However, the number of samples by temperatures varied according to enzyme activity loss. A relative enzyme activity loss of about ~ 75% was set up as the limit for sampling. Nonetheless, when enzyme activity loss exceeded before the end of the sampling time assigned; a minimum of five sampling times was established (whenever possible).

A stratified sampling strategy allows estimating the enzyme activity (UL^− 1^) average at different sampling moments for each temperature. The initial population (time 0) at each temperature, consisted of three batches (L1, L3 and L4) with the same number of vials, containing 2 mL polypropylene screw cap tube (BIOLOGIX) with one millilitre of recombinant enzyme concentrate from each respective batch (L1, L3, L4). Vials of each batch were stored in two square cryopreservation boxes (10 × 10 vials). Each batch constructed constituted a selection stratum for each sampling time.

An Excel spreadsheet was programmed to determine population size (N), varied population sizes were assigned and tested to estimate the mean value in the stratified sampling with proportional allocation, using different levels of precision and confidence, and considering the costs that designs would entail [43]. Finally, the population size calculated was assumed for the three strata (L1, L3 and L4), allowing to estimate the median value of the enzymatic activity of rPOXA1B (UL^− 1^) for each sampling moment, with a precision of 21.8 (UL^− 1^) and 95% confidence.

According to stratified sampling, a simple random sample was taken from each batch at each sampling time, to estimate the average enzyme activity of rPOXA 1B (UL^− 1^) and its corresponding confidence interval (CI). For estimations, we used stratified probability sampling bases with proportional allocation [43].

Sampled vials at each time of the stability study allow to determining enzyme activity (UL^− 1^). Later, the rest of the sample of the same time point and temperature were pool to determine by triplicate apparent *V*_*max*_ and *K*_*M*_, with their respective ANOVA analysis using SPSS 19. To determining *V*_*max*_ and significant differences, a 0.05 significance was established for the temperature range assayed.

### Real-time stability of rPOXA 1B

Real-time stability of rPOXA 1B, was determined by plotting (isotherms) average enzyme activities ± confidence interval (CI) for each sample, at each sampling time. To this object SigmaPlot V11.0., was used. Relative enzyme activity was the percentage of activity recovered at each sampling time, compared to the initial volumetric enzyme activity (100%).

### rPOXA 1B accelerated stability analysis and thermodynamic parameters

The slope from the Ln (*E/E*_*o*_) graph as a function of time (months), based on the enzyme activity (UL^− 1^) analysis of the first-order kinetics for each of the isotherms (243.15, 277.15, 298.15, 303.15, 308.15 and 313.15 K) allows calculating the inactivation rate constant (*k*_*d*_). *E*_*0*_ is the initial enzyme activity (UL^− 1^) and (*E*) is the average enzyme activity (UL^− 1^) for each sampling time. Deactivation energy (*E*_*d*_) was calculated by plotting the Ln (*k*_*d*_) graph obtained for each isotherm versus the inverse of the temperature (1/T) in K, using Arrhenius (Eq. 1).

The enzyme’s half-life (t_1/2_) is the time required for the enzyme to decrease in half its initial activity [31], and by using (Eq. 2) it was calculated.

Thermodynamic parameters ∆H (change in enthalpy of deactivation), (Eq. 3), ∆G (change in Gibb’s free energy of inactivation), (Eq. 4) and ∆S (change in entropy of inactivation), (Eq. 5) for an irreversible inactivation were calculated from the following equations.

1 $$ \mathit{\ln}\left({k}_d\right)=\mathit{\ln}\ (A)-\frac{E_d}{RT} $$

2 $$ {t}_{1/2}=\frac{\mathit{\ln}2}{k_d} $$

3 $$ \Delta  H={E}_d-\mathrm{RT} $$

4 $$ \Delta  G=-\mathrm{RT}\ \ln \left(\frac{k_dh}{k_bT}\right) $$

5 $$ \Delta  S=\frac{\left(\Delta  \mathrm{H}-\Delta  \mathrm{G}\right)}{T} $$

Where: *k*_*d*_ is the deactivation constant, A is the frequency factor, *E*_*d*_ is the deactivation energy, R is the universal gas constant (8.314 J mol^− 1^ K^− 1^), and T is the absolute temperature (Kelvin) [31, 44], *h* is the constant of Plank (6.626× 10^− 34^ J s) and *k*_*b*_ is the constant Boltzmann (1.38 × 10^− 23^ JK^− 1^), [44, 45].

### Determination of laccase activity

Laccase enzyme activity was determined using ABTS (2,-Azino-bis(3-ethylbenzothiazoline-6 sulfonic acid) as a substrate. Into a 100 μL spectrophotometric cuvette 20 mM ABTS and centrifuged supernatant (from 2 to 20 μL, depending on the amount of enzyme present in the sample were added). To complete a final volume of 1 mL 0.1 M citrate buffer (pH 3.0 ± 0.2) was added. One-minute absorbance change resulting from ABTS oxidation at 420 nm was measured [41]. One unit of the enzyme (U) is defined as the quantity of enzyme capable of transforming 1 μmol ABTS substrate per minute, per litre, and was calculated based on (Eq. 6).

6 $$ {UL}^{-1}=\frac{\left(\varDelta E\times {V}_t\right)}{\left(\varepsilon \times d\times {V}_s\right)} $$

Where: *ΔE* corresponds to the difference between final and initial absorbance after 1 min of reaction, *V*_*t*_ refers to the total reaction volume (mL), *Ɛ* refers to the ABTS molar extinction coefficient (M^−1^ cm^−1^) at 420 nm, *d* is the length of the cuvette in cm and V_s_ is the volume of sample (mL) contained in the reaction.

### Apparent enzyme kinetic constants

As the real-time stability study progressed and once laccase activity was quantified, at each sampling moment, the remaining volume of samples (n) were pool (distinguishing by batch, by temperature, by sampling time) to determine the kinetics of the enzyme. For the enzyme kinetic assay ABTS dissolved in 0.1 M citrate buffer, was used as a substrate (concentration between 0.1–3 mM), pH 3.0 ± 0.2. An enzyme solution with an activity of 10 UL^−1^ at 25 °C [46]. All kinetic tests performed in triplicate. The experimental data from each enzyme kinetic were feed to Biomodel software (http://biomodel.uah.es/metab/enzimas/inicio.htm), which allows fitting the experimental data to the Michaelis-Menten equation (Eq. 7) using a non-linear, least-squares based regression. *K*_*M*_ and *V*_*max*_ values previously estimated by the Hanes-Woolf linear regression (Eq. 8), [47] by using SIMFIT software (V7.6.8), [48] used to start the iteration process at Biomodel.

7 $$ {V}_0=\frac{V_{max}\left[S\right]}{K_M+\left[S\right]} $$

8 $$ \frac{\left[S\right]}{V_0}=\frac{\left[S\right]}{V_{max}}+\frac{K_M}{V_{max}} $$

Where: *V*_*0*_ is the initial velocity, *V*_*max*_ is the maximum reaction rate, *K*_*M*_ is the Michaelis constant and [*S*] is the substrate concentration.

### Homology modeling of POXA 1B

Since there is no crystal structure reported for POXA1B from *Pleurotus ostreatus* it was necessary to create a 3D model for the enzyme; then the sequence was processed in the SignalP 5.0 server (http://www.cbs.dtu.dk/services/SignalP/) to identify and eliminate peptide signal amino acids.

Laccase 3D structure was predicted by homology modelling [42, 49–51] using the HHpred, Phyre2 and Swiss-model servers. The resulting model was evaluated by QMEAN [52, 53] and for characteristics of the enzyme’s active site. Saves V 5.0 server allows validating the quality of the modelled structure (https://servicesn.mbi.ucla.edu/SAVES/).

### POXA 1B molecular dynamics simulations at different temperatures

The CuT1 copper and TNC in laccase’s binding pocket were parametrized, by using the metal centre parameter builder (MCPB.py), [54] included in the Amber18 package [55], the partial charges were obtained from the electrostatic calculation by using Gaussian 16 [56]. A complete set of the parameters generated by mcpb.py for cooper ions and their coordination residues are in Supplementary Material S1. All coppers (Cu type I and Cu from TNC) were modelled as Cu^2+^. The hydrogens and protonation state for each residue were carried out with webserver H++ (http://biophysics.cs.vt.edu/) adjusting the PDB file of POXA 1B to pH 3.0. For MD simulations topology files and system, coordinates were prepared using the tleap interface. The system was neutralized with Na^+^ and Cl^−^ and was immersed in a TIP3P water box with 10.0 Å between any atom of the protein and the edge of the box. Additionally, the ff14SB force field was used to model all amino acid residues [57].

To compare the stability of the enzyme at different temperatures, four temperatures were set up [241 K (− 32 °C); 278 K (5 °C); 298 K (25 °C) and 314 K (41 °C)]. Initial energy minimization was carried out, using 50,000 steps of the steepest descent algorithm steps, followed by 10,000 steps of conjugate gradients. The minimized system was gradually warmed up to four stages from 0 to 241 K (stage 1), from 241 to 278 K (stage 2), from 278 to 298 K (stage 3) and from 298 to 314 K (stage 4); using an NVT canonical ensemble and a Langevin thermostat with a 2.0 ps^− 1^ collision frequency and a 2 fs step size Newton equations were integrated.

All simulations were performed with the same cut-off and electrostatic interactions beyond the cut-off distance are ignored. In all simulations, a cut-off of 8.0 Å was employed for both the electrostatics and the van der Waals interactions. The imposition of periodic boundaries on the system during the calculation was controlled at constant pressure with isotropic position scaling and in the production were kept the coordinate output from overflowing the trajectory.

With the results obtained from each of the heating stages, were evaluated several MD stages. The density of each heating step was equilibrated for 400 and 200 ns production and simulated using an isothermal-isobaric (NPT), hydrogen length constrained (SHAKE) assembly. Snapshots of the scenario were taken every 0.2 ns during production (1000 in total). The trajectories obtained for each temperature were analysed using CPPTRAJ [58, 59].

### Principal component analysis

To evaluate movements of the main chains of the system for the four temperatures studied principal component analysis (PCA) was carried out, according to previously described methodology [60]. The covariance matrix was constructed from MD topology and trajectory obtained files. The root mean square (RMS) was adjusted to eliminate rotational/translational movements and preserve internal dynamics, except for hydrogen atoms. The first five principal components corresponding to the first eigenvectors of the covariance matrix were selected. Structural representations of the different modes were constructed in CPPTRAJ and visualized in Visual Molecular Dynamics (VMD). Additionally, for the PCA a superposition of Cα atoms in the MD trajectories using R’s Bio3D package [61] was constructed from which the contribution of each residue was evaluated for the first five main PCAs [59].

## Results

### Pilot study of enzyme stability

The stability pilot assay demonstrated that as the temperature increased, the relative activity of the enzyme decreased. Between 243.15 K (− 30 °C) and 277.15 K (4 °C) there were slight enzyme activity variations at day 30 of the study. At 303.15 K (30 °C) relative enzyme activity was maintained (~ 85%); whereas at 313.15 K (40 °C) and 323.15 K (50 °C), temperatures were destabilizing for the system, with a considerable reduction of the enzyme activity (Table 1).

**Table 1:** Percentage of rPOXA 1B relative activity preserved during 30 days at five theoretical study temperatures

Temperature °C	Temperature K	Time (days)	
		15	30
		% relative enzyme activity	
−30	243.15	95.38	96.74
4	277.15	88.50	95.36
30	303.15	84.76	85.59
40	313.15	74.05	61.30
50	323.15	25.96	13.39

Assuming equal variances for each temperature, a combined variance estimator (an estimator commonly used in parametric tests) was used to obtain an estimate of enzyme activity variability; with an estimate of 96.6 for rPOXA 1B UL^− 1^ standard deviation.

### Real-time stability of rPOXA 1B concentrate

Table 2 illustrates population size (*N*) and sample size (*n*), at the beginning of the study (month 0) and at each sampling time (months). For the theoretical temperatures of 30 and 4 °C averages of 240.98 ± 5.38 K (− 32.55 ± 4.12 °C) and 277.40 ± 1.32 K (4.32 ± 1.22 °C) were obtained, and the 12 months of the study were completed. For the theoretical temperatures of 25, 30, 35 and 40 °C, averages of 297.53 ± 3.88 K (24.99 ± 0.25 °C), 303.27 ± 1.11 K (30.12 ± 1.11 °C), 309.58 ± 0.23 K (36.43 ± 0.23 °C) and 314.79 ± 0.52 K (41.64 ± 0.52 °C) were obtained.

**Table 2:** Sampling distribution carried out for each lot and sampling time

Month	Sampling time	***N***	***n***
1	1	200	16
2	2	184	16
3	3	168	16
4	4	152	16
5	5	136	16
6	6	120	15
8	7	105	15
10	8	90	15
12	9	75	14

*N*: Population size, *n*: sampling size

**Fig. 1:**
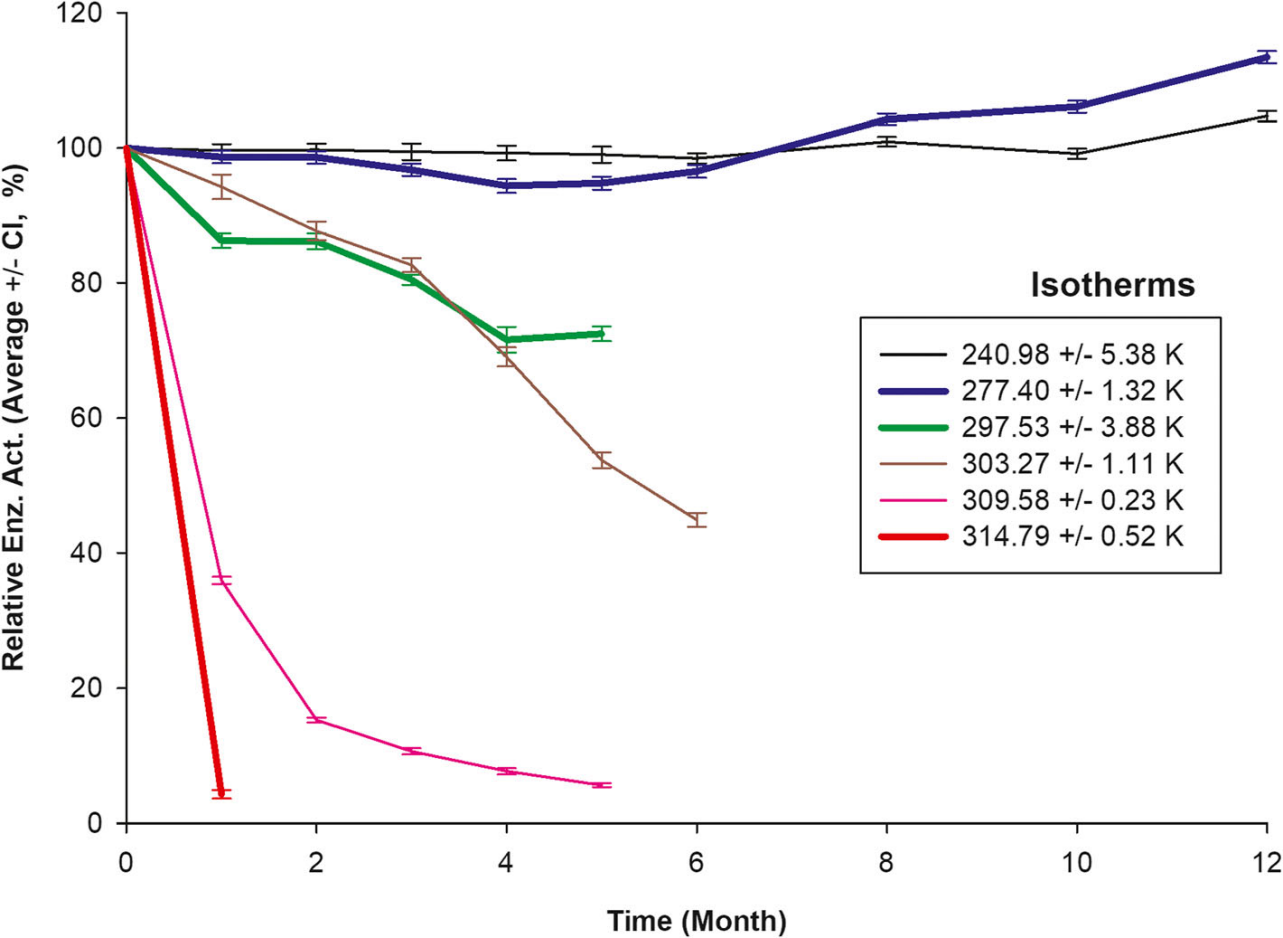
rPOXA 1B real-time enzyme stability study. Percentage of relative enzyme activity isotherms of the L1, L3 and L4 batches average ± CI of vs., time in months for each assayed temperature. The three temperatures with the thickest lines (blue, green and red) are those studied computationally. The enzyme activity (UL^− 1^) was averaged, at each sample, batch and storage temperatures. The average enzyme activity allows estimating the percentage of relative enzyme activity concerning the initial enzyme activity

Figure 1, depicts relative enzyme activity ± confidence interval and in Table 3 rPOXA 1B specific activity (U mg^− 1^) ± confidence interval (CI), as a function of time exposed to different study temperatures. At 240.98 ± 5.38 K, it was observed the activity remained constant throughout the 12 months of the study. At 277.40 ± 1.32 K, a slight increase in enzyme activity percentage was observed after the sixth month of sampling. For the temperatures of 297.53 ± 3.88, 303.27 ± 1.11 and 309.58 ± 0.23 K, a slight gradual reduction in enzyme activity was observed as the temperature increased, maintaining a residual enzyme activity of 75.23, 46.09 and 5.81%, respectively, until the last sample. At 314.79 ± 0.52 K, a considerable loss in relative enzyme activity was observed for the first month of sampling, with a residual enzyme activity of 4.83% (Fig. 1**,** Table 3).

**Table 3:** Specific activity (U mg^−1^ ± CI) during sampling months

**Temperature K**	**240.98 ± 5.38**	**277.40 ± 1.32**	**297.53 ± 3.88**	**303.27 ± 1.11**	**309.58 ± 0.23**
**Temperature °C**	**−32.55 ± 4.12**	**4.32 ± 1.22**	**24.99 ± 0.25**	**30.12 ± 1.11**	**36.43 ± 0.23**
**Time (Month)**	**Specific Activity U mg**^**− 1**^ **± CI**				
1	758.92 ± 8.90	857.23 ± 10.09	745.57 ± 12.32	759.46 ± 16.71	236.74 ± 4.07
2	763.62 ± 7.75	872.88 ± 9.77	737.70 ± 12.11	679.41 ± 11.49	116.74 ± 2.71
3	752.30 ± 9.54	863.89 ± 9.64	663.10 ± 11.70	627.46 ± 10.27	76.87 ± 3.09
4	758.06 ± 9.03	860.29 ± 10.13	587.68 ± 14.01	520.02 ± 10.29	54.15 ± 2.86
5	754.66 ± 9.95	878.27 ± 9.78	567.65 ± 13.30	395.92 ± 9.83	37.65 ± 1.99
6	763.25 ± 6.38	884.82 ± 10.15	563.05 ± 0.0		
8	787.08 ± 6.27	959.30 ± 10.84			
10	759.43 ± 6.30	997.01 ± 13.22			
12	934.81 ± 9.70	1045.58 ± 15.31			

### rPOXA 1B accelerated stability and thermodynamics parameter analysis

**Fig. 2:**
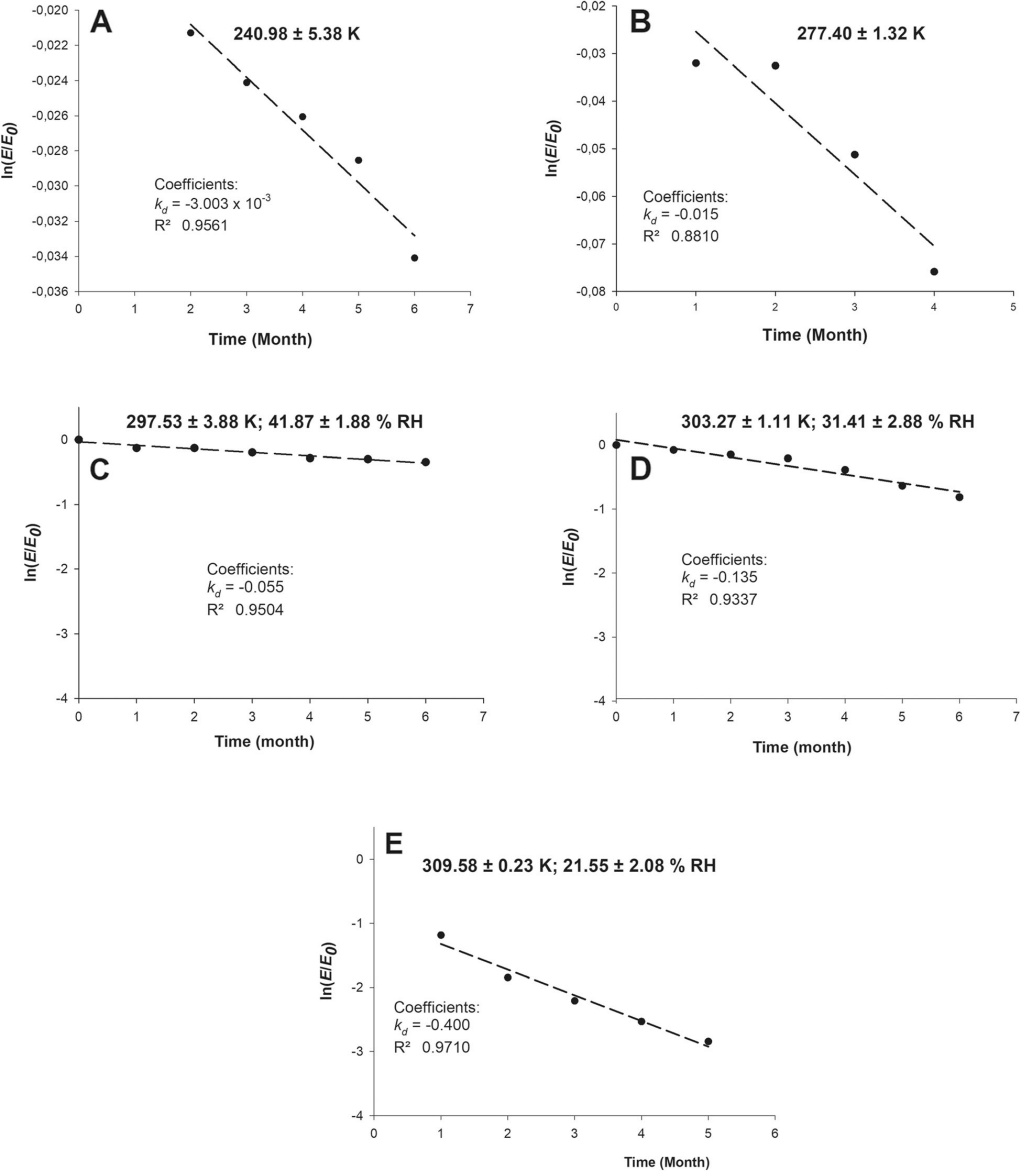
Plot of ln [*E/E*_*0*_] versus time (month) for the calculation of deactivation constant (*K*_*d*_). rPOXA 1B first- order deactivation kinetics at different temperatures A: 240.98 ± 5.38 K (− 32.55 ± 4.12 °C), B: 277.40 ± 1.32 K (4.32 ± 1.22 °C), C: 297.53 ± 3.88 K (24.99 ± 0.25 °C), D: 303.27 ± 1.11 K (30.12 ± 1.11 °C), E: 309.58 ± 0.23 K (36.43 ± 0.23 °C). RH is the average of relative humidity (%) at the study temperature

**Fig. 3:**
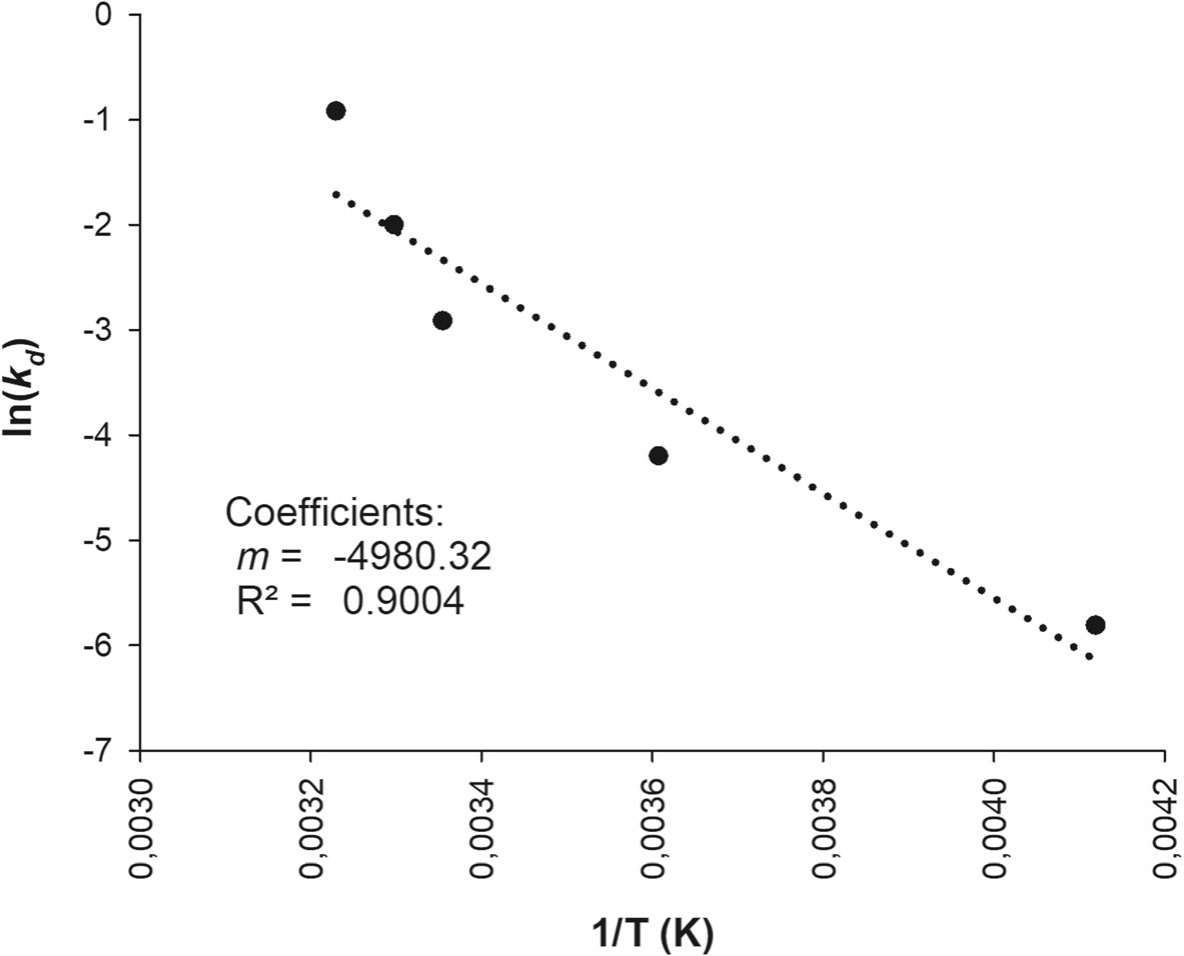
Arrhenius plot to get deactivation energy (*E*_*d*_) calculation of rPOXA 1B exposition temperatures between 240.98 ± 5.38 K (− 32.55 ± 4.12 °C) and 309.58 ± 0.23 K (36.43 ± 0.23 °C)

Accelerated stability calculations were obtained as a function of time, and rPOXA1B deactivation using different exposure temperatures. First-order deactivation kinetic yielded the enzymatic deactivation constant (*k*_*d*_), (Fig. 2**,** Table 4) with R^2^ between 0.9710 and 0.8810. Half-life (t_1/2_) and deactivation energy (*E*_*d*_) (Fig. 3**,** Table 4) were calculated from obtained *k*_*d*_ at different temperatures.

**Table 4:** rPOXA 1B concentrate (enzyme without purification) thermodynamic parameters, calculated during thermal deactivation at study temperatures.

Thermodynamic parameters	Temperature K				
	240.98 ± 5.38	277.40 ± 1.32	297.53 ± 3.88	303.27 ± 1.11	309.58 ± 0.23
	Temperature °C				
	−32.55 ± 4.12	4.32 ± 1.22	24.99 ± 0.25	30.12 ± 1.11	36.43 ± 0.23
***K***_***d***_ (months^−1^)	3 × 10^− 3^	0.015	0.055	0.135	0.400
**t**_**1/2**_ (months)	230.8	46.2	12.6	5.1	1.7
***E***_***d***_ (kJ mol^−1^)	41.40				
**ΔH** (kJ mol^−1^)	39.40	39.10	38.93	38.88	38.83
**ΔG** (kJ mol^−1^)	70.23	77.46	80.04	79.37	78.28
**ΔS** (J mol^−1^ K^− 1^)	−127.94	− 138.30	−138.17	− 133.50	− 127.42

The thermodynamic parameters to identify the stability of rPOXA 1B appears in Table 4. ∆H was constant over the study range, as the ∆H values only decreased by approximately 1.5% with increasing temperature by 70 K; this variation is within the experimental uncertainty. The ∆G showed high and positive values, while ∆S exhibited negative values.

### Effect of different temperatures on (apparent) *V*_*max*_ and *K*_*M*_

Observed apparent *V*_*max*_ and *K*_*M*_ appear in Table 5**.** *V*_*max*_ for enzymes at different temperatures revealed that between 240.98 ± 5.38 and 297.53 ± 3.88 K no variation was observed, whereas it decreased for higher temperatures (309.58 ± 0.23 and 314.79 ± 0.52 K). *K*_*M*_ variation was associated with exposure temperature, increasing as exposure temperature augmented. For both analyses, differences between temperatures were significant (Table 5).

**Table 5:** Apparent *V*_*max*_ and *K*_*M*_ average variation after X time exposure at different temperatures

				Non-linear, least-squares based regression	
Temperature^a^		Time	Number of	V_**max**_ ± SD	K_**M**_ ± SD
(°C)	(K)	(Month)	(pool)	(mM min^**−1**^)	(mM)
−32.55 ± 4.12	240.98 ± 5.38	12	27	0.0102 ± 0.0004	0.0414 ± 0.0046
4.32 ± 1.22	277.40 ± 1.32	12	27	0.0104 ± 0.0005	0.0435 ± 0.0057
24.99 ± 0.25	297.53 ± 3.88	6	18	0.0102 ± 0.0004	0.0466 ± 0.0064
30.12 ± 1.11	303.27 ± 1.11	6	18	0.0104 ± 0.0004	0.0477 ± 0.0076
36.43 ± 0.23	309.58 ± 0.23	4	12	0.0094 ± 0.0005	0.1016 ± 0.0736
41.64 ± 0.52	314.79 ± 0.52	1	3	0.0096 ± 0.0006	0.1433 ± 0.1564

^a^Temperatures at which the samples were stored during the real-time stability study

Kinetic enzymatic reaction conditions were ABTS dissolved in 0.1 M citrate buffer at concentration between 0.1–3 mM, pH 3.0 ± 0.2. An enzyme solution with an activity of 10 UL^− 1^, reaction temperature 25 °C

### Homology model of POXA 1B

Evaluated servers generated different predictions for POXA1B 3D’s protein structure from amino acid sequences. However, among servers, the predictions obtained from HHpred and Phyre2 did not result in a model according to the distribution of copper atoms at the site of the enzyme. Therefore, we decided to evaluate different predicted models using an automated comparative 3D protein modelling server SWISS-MODEL [62, 63]. The SWISS-MODEL template library was searched with BLAST [64] and HHBlits [65].

Templates for POXA 1B modelling and models obtained from 5Z1X (*Cerrena* sp. RSD1), 5E9N (*Steccherinum murashkinskyi*) and 1GYC (*Trametes versicolor*) were evaluated, with identities of 64.12, 62.96 and 62.50%, respectively. The characteristics of the active were evaluated and it was observed the 1GYC template had a more suitable configuration at the active site. The model obtained with the selected template obtain a 0.80 QMEAM and a GMQE of 0.87. Predicted results for the model are in Supplementary Material Figure 1. Validation of the structure was supported by Saves 5.0 model structure scores, which according to Ramachandran’s plot revealed that 88.9% of the residues were in the most favoured region and 10.6% in the additional allowed region (Supplementary Material Figure 2). Verify 3D score was 94.08%, ERRAT score was 88.7734 and the PROVES score was 4.1%.

### Molecular dynamics simulations and PCA analysis

In the active site parametrization, CuT1 copper was coordinated with His^394^, HIS^496^ and CYS^451^. Distance between CuT1 and the TNC was approximately 12 Å. CuT2 was coordinated with HIS^64^, HIS^397^ and a hydroxyl group. CuT3 coppers were coordinated with six histidines (HIS^66^, HIS^109^, HIS^111^, HIS^399^, HIS^450^, HIS^452^) and a hydroxyl group between CuT3훂 and CuT3훃 (Supplementary Material Figure 3).

To evaluate the effect temperature (241 K [− 32 °C], 278 K [5 °C], 298 [25 °C] K and 314 K [41 °C]) had on the enzyme, MD simulations were performed. Simulated system stability at different temperatures was evaluated by calculating the root-mean-square deviation (RMSD) during the simulation time. It was observed that the conformational stability of the systems was maintained during the simulations since the deviation did not exceed 2 Å for all evaluated temperatures (Supplementary Material Figure 4). Furthermore, it was noticed deviations increased as the simulation temperature was raised. Thus, the higher deviations corresponded to 314 K (40.85 °C), (Supplementary Material Figure 4).

**Fig. 4:**
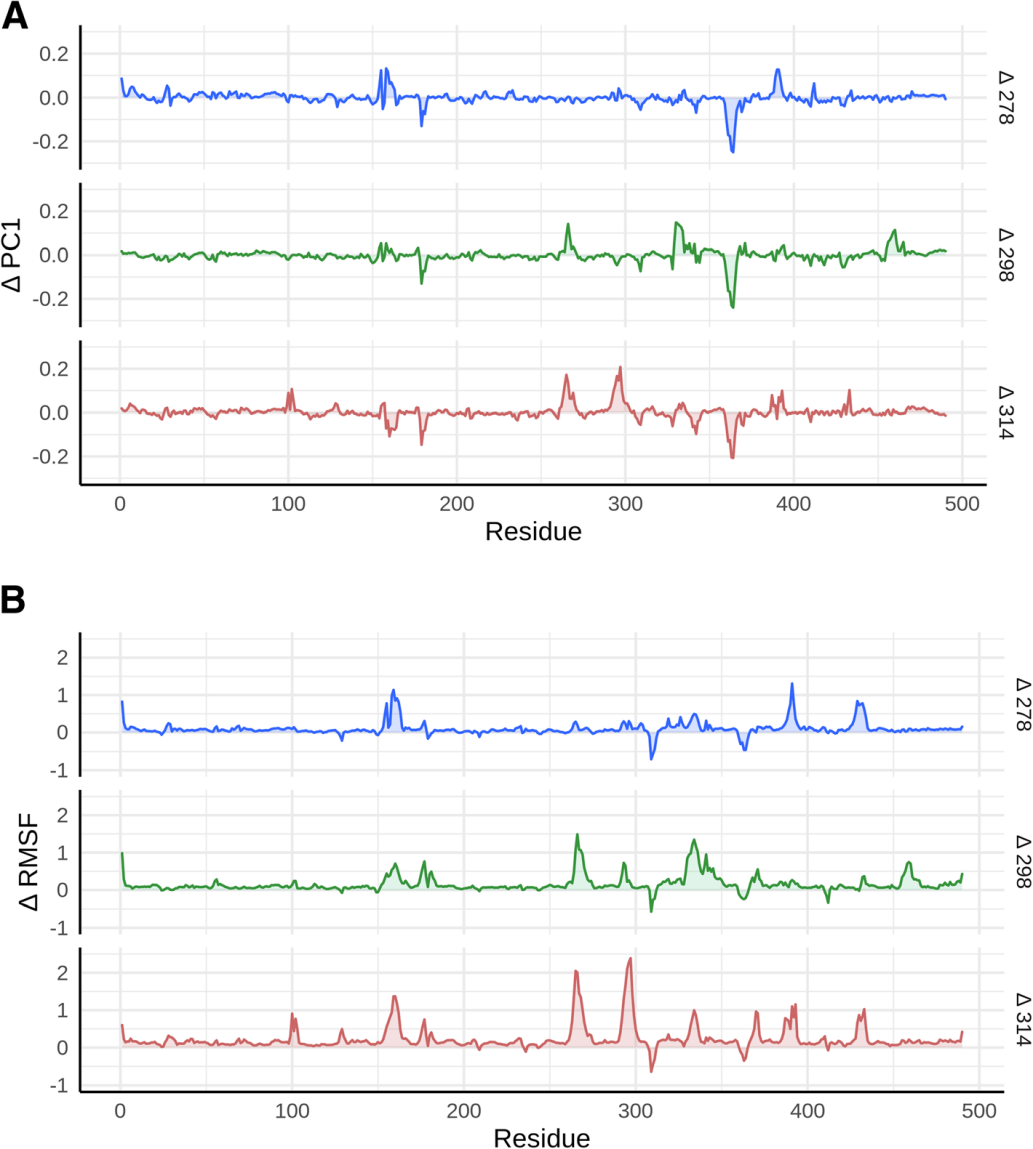
Comparison of the RMSF differences (∆ RMSF) calculated between the different study temperatures against 241 K. In the ∆ RMSFs, a positive value indicates that the residual has fluctuations, and a negative value indicates the opposite A. ∆ RMSF (278–241 K blue; 278–241 K green and 314–241 K red). B. ∆ PC1 (278–241 K blue; 278–241 K green and 314–241 K red). The protein skeleton (C, CA and N), averaged per residue, was used to calculate the RMSF

**Fig. 5:**
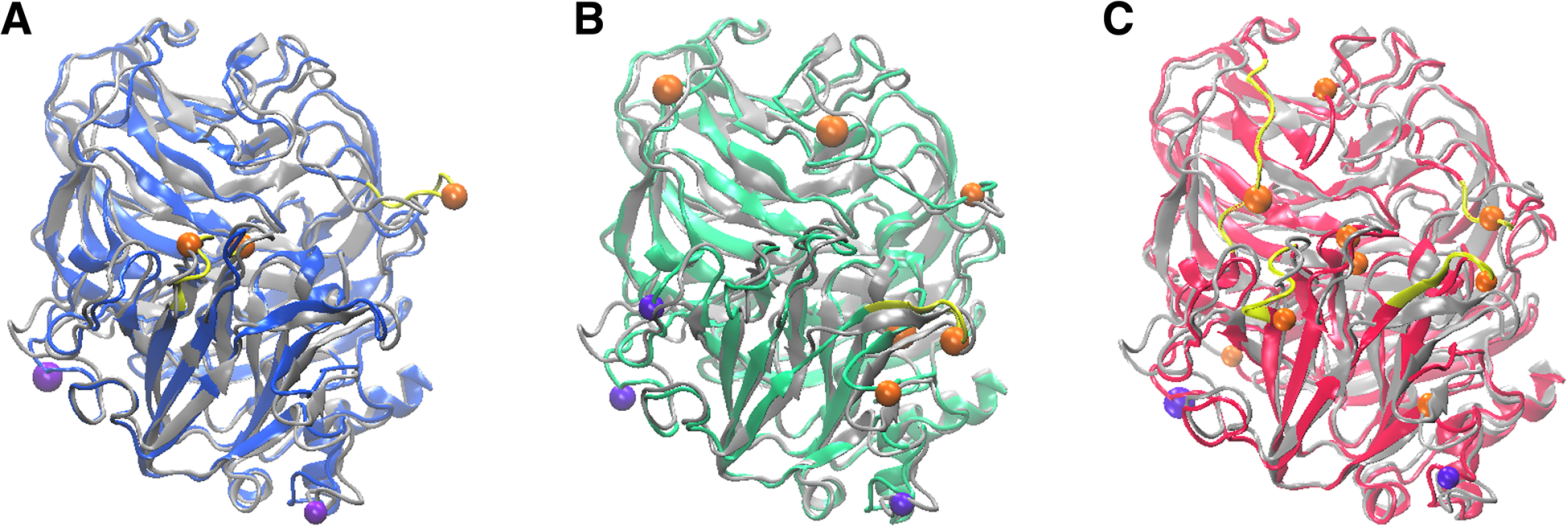
Effect of temperature on residues and regions in the MD simulation of POXA 1B. 3D structure of POXA 1B using Visual Molecular Dynamics (VMD) with the location of the residues or enzyme regions with the large fluctuations. Orange beads and yellow region are residues with the highest fluctuation than violet beads remarket regions. A. 278 K (blue). B. 298 K (green). C. 314 K (red). The 3D structure of 241 K (grey) overlayed with the 3D structures of POXA 1B at others temperatures

The structural changes responsible for the system’s destabilization were determined by calculating the average root mean square fluctuations (RMSF) for each of the simulated temperatures. During the MD simulation, each residue’s fluctuation (RMSF) was analyzed (Supplementary Material Figure 5). In addition, a comparison by residue between the different temperatures an ∆ RMSF was calculated considering as reference the lowest temperature (278–241 K, 298–241 K and 314–241 K), Fig. 4A. The aforementioned facilitated identifying the regions and/or residues leading to fluctuations as a function of temperature in the simulation. In Fig. 4A two negative peaks were observed for ∆ RMSF at 278, 298 and 314 K corresponding to LYS^309^ and ALA^363^ amino acids (Violet beads - Fig. 5), which indicate the fluctuation was less in comparison with the temperature at 241 K. Moreover, ∆ RMSF 298 K an additional negative peak corresponding to residue SER^412^ was observed. On the other hand, it was recognized the number of residues with the biggest fluctuations augmented with increasing temperature.

For ∆ RMSF 278 K, in Figs. 4A and 5A, it was observed residues LEU^159^, ALA^391^ and ASP^429^ (Orange beads – Fig. 5A) presented the highest fluctuations, and the residues in proximity to LEU^159^ and ASP^429^ generated fluctuating regions. Figures 4A and 5B for ∆ RMSF 298 K residues with high fluctuations were THR^160^, ASP^266^, GLU^293^, ALA^334^, ASP^341^ and LEU^459^ (Orange beads – Fig. 5B), only amino acids close to ALA^334^ conformed a fluctuating region (Yellow region - Fig. 5B). In Figs. 4A and 5C**,** for ∆ RMSF 314 K residues with high fluctuation increased, where ASP^101^, THR^160^, GLY^265^, ASN^297^, ALA^334^, GLY^370^, ALA^391^, PRO^393^ and ASP^433^ were of the greatest fluctuation (Orange beads – Fig. 5). Likewise, residues close to THR^160^, ASN^297^, ALA^334^ and ASP^433^ generated fluctuating regions (Yellow region - Fig. 5C).

Residues and regions with ∆ RMSF that increased fluctuation in a progressive manner, according to the increase in temperature, were identified as ASP^266^, with its region in the vicinity between SER^264^ - PHE^269^; and ASN^297^ with its region in the vicinity between GLN^293^ - PRO^298^. Standard deviation calculation among ∆ RMSF revealed the residues with standard deviations greater than 0.5 Å (cut off point) corresponded to the same previously identified residues, including the additional residues ALA^391^ and PRO^393^.

RMSF and PCA supported residue contribution to the system’s atomic fluctuation variations. The PCA was performed using C훂 position co-variance to describe the global movements of rPOXA 1B at different temperatures employing the first principal components (PC).

Furthermore, to make a comparison by residue the first ∆ PC was calculated considering as reference the lowest temperature; for instance, 278–241 K, 298–241 K and 314–241 K. In Fig. 4B**,** ∆ PC1 are depicted for 278–241 K, 298–241 K and 314–241 K, which included previously observed residues and regions in the ∆ RMSF. For ∆ PC1 278 K the included residue was ALA^391^ and the included regions were PRO^155^ - THR^160^ and THR^360^ - ASP^365^. For ∆ PC1 298 K the residue was ASP^266^ and the regions ALA^330^ - ALA^334^, THR^360^ - ASP^365^ and ASP^458^ - GLY^460^. For ∆ PC1 314 the residues were GLU^101^, THR^160^, PRO^393^, ASP^433^ and the regions ASN^263^ - PRO^267^, GLN^293^ - PRO^298^, THR^360^ - ASP^365^. PC1 represented 15.4, 15.9, 11.8 and 18.6% variance proportion for 241 K (− 32 °C), 278 K (5 °C), 298 K (25 °C) and 314 K (41 °C), respectively.

∆ PC2 and ∆ PC3 were calculated similarly to ∆ PC1 (Supplementary Material Figure 7) and included the missing residues that showed fluctuation in the ∆ RMSF and were not represented in ∆ PC1. The first five components accounted for approximately 41% of the variations at 241 K (− 32.15 °C), 278 K (4.85 °C) and 314 K (40.85 °C) and ~ 36% of the variations at 298 K (24.85 °C) (Supplementary Material Figure 8).

## Discussion

### Pilot study of rPOXA 1B stability

The pilot study demonstrated temperatures ranging between − 30 °C (243.15 K) and 40 °C (313.15 K), were appropriate to perform stability studies since at 40 °C (313.15 K) residual activity was of 61.30% **(**Table 1**)**. In previous studies [40], rPOXA 1B demonstrated stability between 10 °C (283.15 K) and 50 °C (323.15 K); the temperature at which relative enzyme activity started to decrease. However, in that study, the exposure lasted only one hour. The pilot study demonstrated the negative effect of exposure time to elevated temperatures. The highest activity loss occurred at 50 °C during the first 15 days, with only 25.9% of the residual enzyme activity recovered, and then at 30 days, it further decreased to 13.2% relative enzyme activity (Table 1).

Prolonged exposure to elevated temperatures induces conformational changes that affect enzyme 3D structure [66]. Nevertheless, this is not common; it is more frequent during short time exposures. Li et al., (2017) evaluated thermal stability at 35 and 55 °C using commercial laccase (Lac), (Sigma, China). In this study, the authors found a relative enzyme activity loss of about 20% when the enzyme exposed for nine hours at 35 °C. Activity loss was about 60 and 100%, when the enzyme exposed during 1 and 9 h, respectively, at 55 °C, (323.15 K) [67]. The authors did not specify the origin of the enzyme, which is a crucial factor, because enzyme stability varies depending on origin and structure.

The sampling design proposed in this work allowed us to construct of a source population from which samples were randomly taken in each of the months (sampling moments). Henceforth in each case, the mean value of enzyme activity was always estimated with the same reliability and precision. The stratified sampling strategy made it possible to control data variations introduced because of using different batches, reducing the sampling error when estimating the enzyme activity mean values.

### Analysis of real-time stability of rPOXA 1B concentrate

Proteins are generally more stable at freezing or refrigeration temperatures [24]. Nonetheless, stability depends on the enzyme’s unaltered structure, hence its biological function. Therefore, non-covalent interactions, such as Van del Waals, hydrophobic-, electrostatic-, and hydrogen-bonds are important [68, 69].

In this work at − 32.55 ± 4.12 °C (240.98 ± 5.38 K), we observed that relative enzyme activity was high during the 12 months of the study (101.16%), (Fig. 1**,** Table 3). Eichlerová et al., (2015) described freezing laccase supernatant obtained from *Trametes versicolor* in liquid nitrogen (− 198 °C, 75.15 K), previous to − 80 °C (353.15 K) storage, generated the highest enzyme activity loss in comparison to direct storage at − 80 °C (353.15 K), [70]. Slow freezing processes produced ice crystals, whereas rapid freezing forms small and slender crystals in the protein’s interfacial area, facilitating its denaturation [71].

Different investigations have analyzed the stability of laccase at freezing temperature, but very few (to the best of our knowledge) have evaluated exposure times longer than 12 months. Zhang et al., (2014) studied the residual activity of freeze-dried laccase from *T. versicolor* (Sigma-Aldrich) during 2 years of storage at − 20 °C (253.15 K), finding a residual enzyme activity of 14.6% [72].

Refrigeration temperature is frequently to evaluate enzyme stability [31, 73]. At 4.32 ± 1.22 °C (277.40 ± 1.32 K) rPOXA1B displayed a relative residual enzyme activity of 115.81% and a residual specific activity of 1045.58 ± 15.31 Umg^− 1^; which implies it was stable during the 12 months of the study **(**Fig. 1**)**. An increase in relative residual enzyme activity is infrequent, yet, Has-Schön et al., (2005) studied peroxidase (E.C. 1.11.1.) stability of a crude *Picea abies* extract at 4 °C (277.15 K) for 30 days and found a relative residual enzyme activity of ~ 190%. These variations are attributable to possible changes in the enzyme’s native environment or the presence of substances that could have acted as activators [74].

In contrast Zhang et al., (2020) obtained a residual enzyme activity of only 10 ± 1.1% after 30-day storage at 4 °C (277.15 K), using *Trametes versicolor* commercial laccase (Sigma-Aldrich), [31]. Bagewadi et al., (2017) monitored *Trichoderma harzianum* HZN10 laccase fining a 45% enzyme activity loss in only 8 days of storage at 4 °C (277.15 K), [75]. Bou-Mitri and Kermasha, (2018) evaluated *Coriolus hirsutus* laccase, finding that after 4 weeks of storage at 4 °C (273.15 K) the residual enzyme activity was 8.5% [73]. rPOXA1B relative residual stability results in this study demonstrated an enzyme with high stability, exceeding that of other laccases, which makes it a promising enzyme for high scale use.

In the present study at 24.99 ± 0.25 °C (240.98 ± 5.38 K), 30.12 ± 1.11 °C (303.27 ± 1.11 K) and 36.43 ± 0.23 °C (309.58 ± 0.23 K) a reduced residual activity was observed with increasing temperature and exposure time, obtaining at the end of the study a relative enzyme activity from 76.43, 46.09 and 5.81%, respectively for each temperature (Fig. 1).

Enzyme denaturation at elevated temperatures is associated with an increase in the kinetic energy of the system, which generates conformational fluctuations despite between 25 °C (298.15 K) and 100 °C (373.15 K) the hydrophobicity appears to remain constant compared to other properties [32]. At 24.99 ± 0.25 °C (297.53 ± 3.88 K) and 30.12 ± 1.11 °C (303.27 ± 1.11 K) the residual enzyme activities were similar until the third month of exposure **(**Fig. 1**)**, but in subsequent sampling. At 30.12 ± 1.11 °C (303.27 ± 1.11 K) the enzyme activity decreased rapidly; an effect that could be due to irreversible thermal denaturation [76]. However, the stability of rPOXA 1B was higher than that reported for other laccases. Ahn et al., (2007) evaluated commercial laccase from *T. villosa* (Novo Nordisk - Danbury, CT), finding that storage for 30 days at 25 °C (298.15 K) generated an exponential deactivation of the enzyme, with a loss of residual relative enzyme activity of 93% [77]. On the other hand, Nadar et al., (2019) evaluated commercial laccase from *T. versicolor* (Sigma-Aldrich), finding that after 18 days at 30 °C (303.15 K) only 30% residual activity was obtained [78].

In the present study, we found that at 41.64 ± 0.52 °C (314.79 ± 0.52 K) a considerable decrease in relative enzyme activity was observed, with a recovery of only 4.83% during the month of exposure, whereas in the pilot study relative residual enzyme activity at the same temperature was of 61.30% (Table 1**,** Fig. 2). In drug stability studies, lower concentrations generally support longer storage times [24]. This concentration effect also applies to biomolecules, since at high concentrations and temperatures enzymes are thermodynamically unstable and prone to form aggregates, due to hydrophobic groups exposure [79]. The difference between specific activity among concentrates used in the pilot study (~ 164.62 Umg^− 1^) and the stability study (758.71 ± 8.90 Umg^− 1^), demonstrates the number of enzyme units per milligram of protein was highest in the stability study. Tung et al., (2018) evaluated FIP-fve protein stability obtained from *Flammulina velutipes* at high and low concentrations during 1 min at 100 °C, recovering 16.4 and 85.0% of protein respectively. However, at 4 °C (277.15 K) no changes were observed, recovering 100% of the protein regardless of the concentration used, yet at − 30 °C (303.15 K) protein loss was slightly lower when the concentration was high [80].

### rPOXA 1B accelerated stability and thermodynamics parameter analysis

The *K*_*d*_ for evaluated temperatures ranged between 3 × 10^− 3^ and 0.400 months^− 1^, with a concomitant increase with the rise in temperature exposure **(**Table 4**)**. This tendency has been previously observed due to enzyme and protein inactivation [78, 81]. *K*_*d*_ was used to calculate the half-life (t_1/2_), revealing that rPOXA 1B at − 32.55 ± 4.12 °C (240.98 ± 5.38 K) and 4.32 ± 1.22 °C (277.40 ± 1.32 K) was stable for at least for 230.7 and 46.2 months, respectively (Table 4).

The half-life (t_1/2_) is an economically important factor since it allows to establish (according to conditions) expiration and storage dates for industrial or environmental applications, forasmuch as the higher the half-life (t_1/2_) the higher the thermostability [82].

Certain reported t_1/2_ values for other laccases have been lower. Yadav et al., (2018) evaluated recombinant small laccase rSLAC from *Streptomyces coelicolor* A3(2) produced in *P. pastoris* and obtained a t_1/2_ of 60, 32 and 10 h at 60 °C (333.15 K), 70 °C (343.15 K) and 80 °C (353.15 K), respectively [83]. In contrast, using commercial *T. versicolor* (Sigma–Aldrich) laccase, Zhang et al., (2020) obtained a t_1/2_ of 7.04, 5.63 and 5.38 h at 30 °C (303.15 K), 35 °C (308.15 K) and 45 °C (318.15 K), respectively [31].

*E*_*d*_ expresses thermal stability [78]. For rPOXA 1B an *E*_*d*_ of 41 KJ mol^− 1^ was obtained (Fig. 3, Table 4), a value that is within the deactivation energy ranges reported by other authors. Nadar and Rathod, (2019) obtained 38.25 kJ mol^− 1^ using commercial *T. versicolor* (Sigma–Aldrich) laccase, Maurya et al., (2020) attained 34.97 kJ mol^− 1^ also using commercial *T. versicolor* laccase (Sigma–Aldrich), [84] and Ahn et al., (2007) obtained 25 kJ mol^− 1^ using *Trametes villosa* laccase (Novo Nordisk - Danbury, CT), [77].

To the best of our knowledge POXA1B from *P. ostreutus E*_*d*_ has not been reported. However, the results herein obtained (*E*_*d*_ = 41 KJ mol^− 1^) confirm high stability for rPOXA 1B, because the highest energy is required to overcome the enzyme’s inactivation barrier. It is known, changes in protein folding generate less organized and unstable molecules, due to the disruption of relatively weaker non-covalent bonds [85].

*E*_*d*_ is directly associated with enthalpy (∆H), a thermodynamic parameter that expresses the total energy associated to enzyme denaturation. Large and positive *E*_*d*_ and ΔH values represent the high thermostability of the enzyme [86]. Positive ∆H values indicate that thermal deactivation is an endothermic reaction [76, 87], where the higher the ∆H value is, the highest the energy required to break the stabilizing bonds in a thermal inactivation of the enzyme [83]. Similar tendencies have reported for laccases and other enzymes [23, 76, 83]. Negative entropy values (∆S) (Table 4) indicate the system has high stability, highest compactness and higher resistance to the thermal inactivation process [45], therefore a reasonable thermostability inside the temperature range studied. Thermal enzyme denaturation also results in the opening of the enzyme structure, accompanied by an increase in the degree of disorder and randomness of the enzyme [86].

A very low or negative ∆G indicate spontaneous reactions and low stability [87]; a positive ∆G indicates resistance to denaturation, i.s. increased thermostability [88]. According to Agbo et al. (2017) an increase in ∆G when temperature raises, means high temperatures thermal stability [76]. However, such a tendency is rather uncommon. For *S. coelicolor* rSLAC laccase, a positive ∆G obtained without exhibit that trend [83]. Similar results were obtained by Filatova et al., (2019) using staphylolytic enzymes (Ply187AN-KSH3b and 2638aR), [23], which demonstrates data here obtained is in line with other investigations. ∆G is considered a more reliable stability indicator than ∆H and ∆S because it includes both enthalpic and entropic contributions [87, 88].

### Effect of exposure to different temperatures on apparent *V*_*max*_ and *K*_*M*_

Table 5 shows that *V*_*max*_ estimated from samples previously preserved between 240.98 K (− 32.55 °C) and 303.27 K (30.12 °C) is constant, suggesting the enzyme’s reaction time was not affected, although at 303.27 K (30.12 °C) amplitude in the standard deviation was observed. At 309.58 K (36.43 °C) and 314.79 K (41.64 °C) the slight decrease in *V*_*max*_ could be explained by the effect of temperature on the enzyme. Michaelis-Menten (*K*_*M*_) constant explains the affinity of an enzyme for a substrate, and the higher the *K*_*M*_, the lower the affinity of the enzyme for the substrate, herein ABTS [84, 89]. In the present study average *K*_*M*_ and SD values progressively increased as temperature increased [240.98 K (− 32.55 °C) and 314.79 K (41.64 °C)]. The effect temperature has on *K*_*M*_ in other investigations has shown that *K*_*M*_ remains constant and without significant differences between 0 and 25 °C. Nonetheless, it presents a distinct threshold with an increase that can be significant at temperatures above 30 °C [90].

### Homology modeling of POXA 1B

Sequence similarity is not the only factor determining 3D structure precision generated by homology. The minimum limit of sequence similarity in homology modelling should be 25% [91]; however, as a general rule, two sequences are homologous if they are more than 30% identical over their entire length. Notwithstanding, homologous sequences that share more than 40% identity are considered functionally similar [92]. In the three templates evaluated, the identity value was greater than 60%. QMEAN and GMQE scores were close to 1, suggesting the proposed model was of high quality and presented a high functional homology.

Based on the residue percentage in central regions in Ramachandran’s plot, POXA 1B 3D structure had a high stereochemical quality (Supplementary Material Figure 1). Verify score value suggested that 94.08% of the 3D modelled atoms were compatible with the sequence. Furthermore, the ERRAT score was adequate. Prognosticated protein models with a quality factor > 50% indicate the models by homology are stable and reliable [93], and the PROVES score was accepted, as it did not exceed 5%.

Our group previously reported a POXA 1B model using as template 1GYC [42]. Nevertheless, it was decided to improve and fine-tune the model using the semiautomatic MCPB.py Amber18 tool for molecules with metallic ion parametrization, since metallic complexes can have various modes of coordination [94], which are important for molecular dynamics analysis.

### Molecular dynamics simulations and principal components analysis

RMSD determines the difference between the main chains of a protein from its initial to its final structural conformation, through the analysis of the deviations produced during the simulation (the minor deviation suggests higher stability of the system), [95]. RMSD calculation demonstrated the stability of the system at different temperatures was maintained during the simulation. Even though the RMSD deviation was higher with increasing temperatures, it remained low, stabilizing at an average of 0.96, 1.06, 1.26 and 1.20 Å for 241 K (− 32.15 °C), 278 K (4.85 °C), 298 (24.85 °C) K and 314 K (40.85 °C), respectively.

Flexibility changes in a protein due to residue fluctuation can destabilize the system [59], where high Cα fluctuations are due to structural changes or free movements in the protein’s backbone [96]. With the MD results, an RMSF and PCA analysis were implemented, to elucidate the effects an increase in temperature would have on the enzyme.

RMSF and PCA analyses complemented each other since PCA represent the residues and identified regions, which are similar in the RMSF analysis. However, to identify the differences in fluctuations among temperatures, ∆ RMSF was evaluated using fluctuations of 241 K (− 32.15 °C) as the base temperature. The negative peaks observed in ∆ RMSF analysis for LYS^309^ and ALA^363^ remained constant during exposure to different temperatures, indicating the stiffness of the system for those residues. LYS^309^ is found on a surface hairpin far from the active site, in the inter-dominium (D2-D3) region. ALA^363^ is found in D3, where the stiffness can be associated with strong interactions, generated by surrounding residues [97]. In contrast to the stiff residues, it was observed residues and flexible regions increased as the temperature was raised (Figs. 4A, 5). Flexible regions specify atom movements of the protein structure, as well as weak interaction sites or regions of the enzyme [98]. Therefore, an increase of the flexible regions demonstrates loss of interactions or bonds that maintain the structure stiff.

ASP^266^, ASN^297^ and nearby regions progressively increased the fluctuation with an increase in temperature (Figs. 4A, 5), shifting from a region with low fluctuation to one with high fluctuation between 278 and 314 K. Both residues, (ASP^266^, ASN^297^) are characterized by being hydrophilic and the nearby regions are composed of hydrophilic residues and residues of intermediate polarity. Hydrophilic residues are more fluctuating than hydrophobic [99]. Moreover, during MD simulations it is usual to observe an increase in flexibility in the hydrophobic regions. Furthermore, ASP^266^ and ASN^297^ residues are located in regions without a defined secondary structure, such as loops or coils; corresponding to regions of the highest fluctuations in any protein [100, 101]; as was observed during the analyses.

The region between LEU^159^ and VAL^162^ residues (Fig. 4A, 5) showed fluctuations in the three ∆ RMSF analysis (278–241, 298–241 and 314–241 K). However, the region with the highest fluctuations was LEU^159^ and THR^160^, which exhibited a higher fluctuation at 278 and 314 K; demonstrating temperature can increase or decrease residue fluctuations. Residue 162, which is not always VAL, has been studied in computational models for *Aspergillus oryzae* (PM1 and 7D5), [4] and *Coriolopsis trogii* [102] laccases, finding this residue is of great importance because it delimits the substrate-binding pocket and is characterized by having a wide network of hydrophobic interactions near CuT1.

The enzyme kinetic stability depends on the delicate balance between the flexibility and rigidity of its active site. The greater the stiffness of the active site, the greater the kinetic stability is [103]. The residues conserved and coordinated with copper ions obtained very low RMSF values, so they were not reported in the PCA. However, the fluctuation of PRO^393^ adjacent residue to HIS^394^-(CuT1) could have generated instability in the system (at 314 K, 40.85 °C). Weakening of copper coordination or subtle rearrangements in the coordination sphere of CuT1 leads to alterations in the interconnection of residues, which could destabilise the entire active site structure resulting in loss of enzymatic activity [104].

Results demonstrated that experimental data (real-time stability studies) was supported by computational (molecular dynamics) since the MD simulated temperatures had high fluctuation regions that could influence the stability of the system, caused by high-temperature exposure, such as 314 K (40.8 °C). In the same way, the application of this computational approach can be used in other enzymatic systems by applying the same described protocols.

## Conclusions

The stratified sampling allows to established population (N) and sample (n) size. The samples at each point in time (months) were random. This approach always allowed us to estimate the median value of the enzyme activity (UL^− 1^) with the same confidence (95%) and precision (21.8 UL^− 1^). Besides, it allows controlling the variation within the results since sampling was carried out from different batches, decreasing sampling error. This statistical approximation done in this research could be considered in other enzymes stability studies. We demonstrated that when rPOXA 1B concentrate (impure and without preservatives agents) stored at − 32.55 ± 4.12 °C (240.98 ± 5.38 K), 4.32 ± 1.22 °C (277.40 ± 1.32 K) or 24.99 ± 0.25 °C (297.53 ± 3.88 K) is stable with t_1/2_ of 230.8, 46.2 and 12.6 months, respectively, exceeding reported storage time for other laccases. rPOXA 1B kinetic and thermodynamic parameters based on *E*_*d*_ (41.40 KJ mol^− 1^) and low K_M_ and *V*_*max*_ variations between − 32.55 ± 4.12 °C (240.98 ± 5.38 K) and 24.99 ± 0.25 °C (297.53 ± 3.88 K); as well as ∆H, ∆G and ∆S values, indicates the enzyme concentrate stability at different temperatures; making it even more attractive for industrial and environmental application. For POXA 1B active centre parametrisation, four coppers coordinated with the HIS^394^, HIS^496^, CYS^451^, HIS^64^, HIS^397^, HIS^66^, HIS^109^, HIS^111^, HIS^399^, HIS^450^, HIS^452^ residues; like the residues reported that coordinates with copper for other laccases. In general, at temperatures between 278 (4.85 °C) and 314 K (40.85 °C) (observed in the MD, using the ∆ RMSF), all fluctuating residues were located at undefined secondary structure regions, such as loops or coils. Likewise, the number of residues with high fluctuations increased with temperatures changing from three residues (LEU^159^, ALA^391^ and ASP^429^) at 278 K (4.85 °C) to six residues (THR^160^, ASP^266^, GLU^293^, ALA^334^, ASP^341^ and LEU^459^) at 298 K (24.85 °C) and nine residues (ASP^101^, THR^160^, GLY^265^, ASN^297^, ALA^334^, GLY^370^, ALA^391^, PRO^393^ and ASP^433^) at 314 K (40.85 °C). Real-time stability analyses and MD confirmed that the increase in temperature starkly affects POXA 1B activity. Temperature increases the fluctuations in hairpin, coils or loops; regions that mainly contain hydrophilic or intermediate polarity amino acids (ASP^266^ and ASN^297^), favouring the long-term exposure of amino acids or hydrophobic zones that maintain the rigidity of the structure, resulting in irreversible inactivation of POXA 1B. At 314 K (40.85 °C), the PRO^393^ residue next to HIS^394^ (3.91 Å distance) increased the fluctuation and could result in the active site instability through alteration of the HIS^394^-CuT1 coordination. This study identified temperatures (under assay conditions) for optimal long-term storage of rPOXA 1B concentrate; allowing the construction of a model based on experimentally assayed temperatures and using the Arrhenius equation to predict the enzyme’s half-life (t_1/2_) at temperatures ranging between 240.98 K (− 32.55) and 309.58 K (36.43 °C). Our results are crucial to the technology transfer related to rPOXA 1B concentrate production at an industrial scale. Also, our findings help companies to decide to commercialise rPOXA 1B based on its multiple applications. Finally, our research group will continue the studies, using the pure enzyme and the addition of non-toxic preservatives to prolong the t_1/2_ further.

## Supplementary Information

**Additional file 1.**

## Data Availability

The raw data supporting the conclusions of this article will be may available by the authors, without undue reservation.

## Abbreviations

MCOs: Blue multi-copper oxidases
POXA 1B: *Pleurotus ostreatus* laccase
rPOXA 1B: Laccase recombinantly expressed in *P. pastoris*
TNC: Trinuclear copper center
SD: Standard deviation
(t_1/2_): Half-life
k_*d*_: Inactivation rate constant
E_*d*_: Deactivation energy
∆H: Change in enthalpy of deactivation
∆G: Change in Gibb’s free energy of inactivation
∆S: Change in entropy of inactivation
ABTS: 2,-Azino-bis (3-ethylbenzothiazoline-6 sulfonic acid)
MCPB.py: Metal center parameter builder based in python
RTS: Real-time stability
AS: Accelerated stability
MD: Molecular Dynamics
NVT: Canonical ensemble
NPT: Isothermal–isobaric ensemble
PCA: Principal component analysis
PC: Principal Component
VMD: Visual Molecular Dynamics
*V*_*max*_: Maximum velocity
*K*_*M*_: Michaelis-Menten constant
*N*: Population size
*n*: Sample size
RMS: Root mean square
RMSF: Root mean square fluctuation
RMSD: Root mean square deviation
ps: Picoseconds
fs: Femtoseconds
ns: Nanoseconds
