# Supplementary material for: Recombinant laccase rPOXA 1B real-time, accelerated and molecular dynamics stability study
Source: BMC Biotechnol. 2021 Jun 4;21:37. doi: 10.1186/s12896-021-00698-3 (PMC8178886; doi:10.1186/s12896-021-00698-3)
Supplement: Supplementary file 1 — Additional file 1. [file 12896_2021_698_MOESM1_ESM.docx]

Supplementary Material

**Recombinant laccase rPOXA 1B real-time, accelerated and molecular dynamics stability study**

Leidy D. Ardila-Leal ^1^, Pedro A. Monterey-Gutiérrez ^2^, Raúl A. Poutou-Piñales ^1^*, Balkys E. Quevedo-Hidalgo ^3^*, Johan F. Galindo ^4^*, Aura M. Pedroza-Rodríguez ^5^

^1^ Laboratorio de Biotecnología Molecular, Grupo de Biotecnología Ambiental e Industrial (GBAI). Departamento de Microbiología. Facultad de Ciencias. Pontificia Universidad Javeriana (PUJ). Bogotá, D.C., Colombia.

^2^ Programa de Maestría y Doctorado en Educación Matemática. Vicerrectoría Académica. Universidad Antonio Nariño, Bogotá, D.C., Colombia.

^3^ Laboratorio de Biotecnología Aplicada, Grupo de Biotecnología Ambiental e Industrial (GBAI). Departamento de Microbiología. Facultad de Ciencias. Pontificia Universidad Javeriana (PUJ). Bogotá, D.C., Colombia.

^4^ Departamento de Química, Universidad Nacional de Colombia, Bogotá, D.C., Colombia.

^5^ Laboratorio de Microbiología Ambiental y de Suelos, Grupo de Biotecnología Ambiental e Industrial (GBAI). Departamento de Microbiología. Facultad de Ciencias. Pontificia Universidad Javeriana (PUJ). Bogotá, D.C., Colombia.


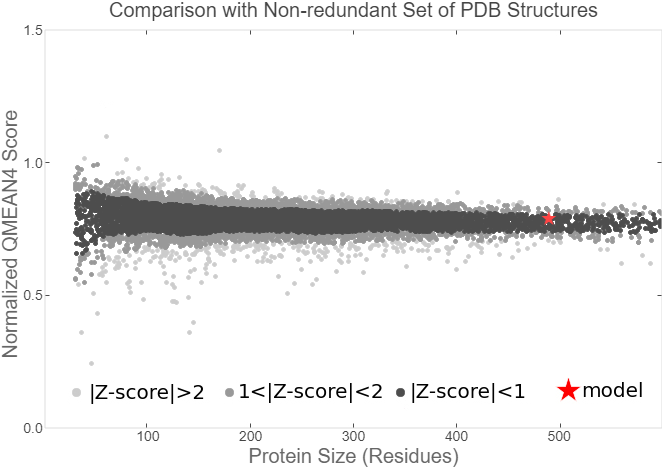

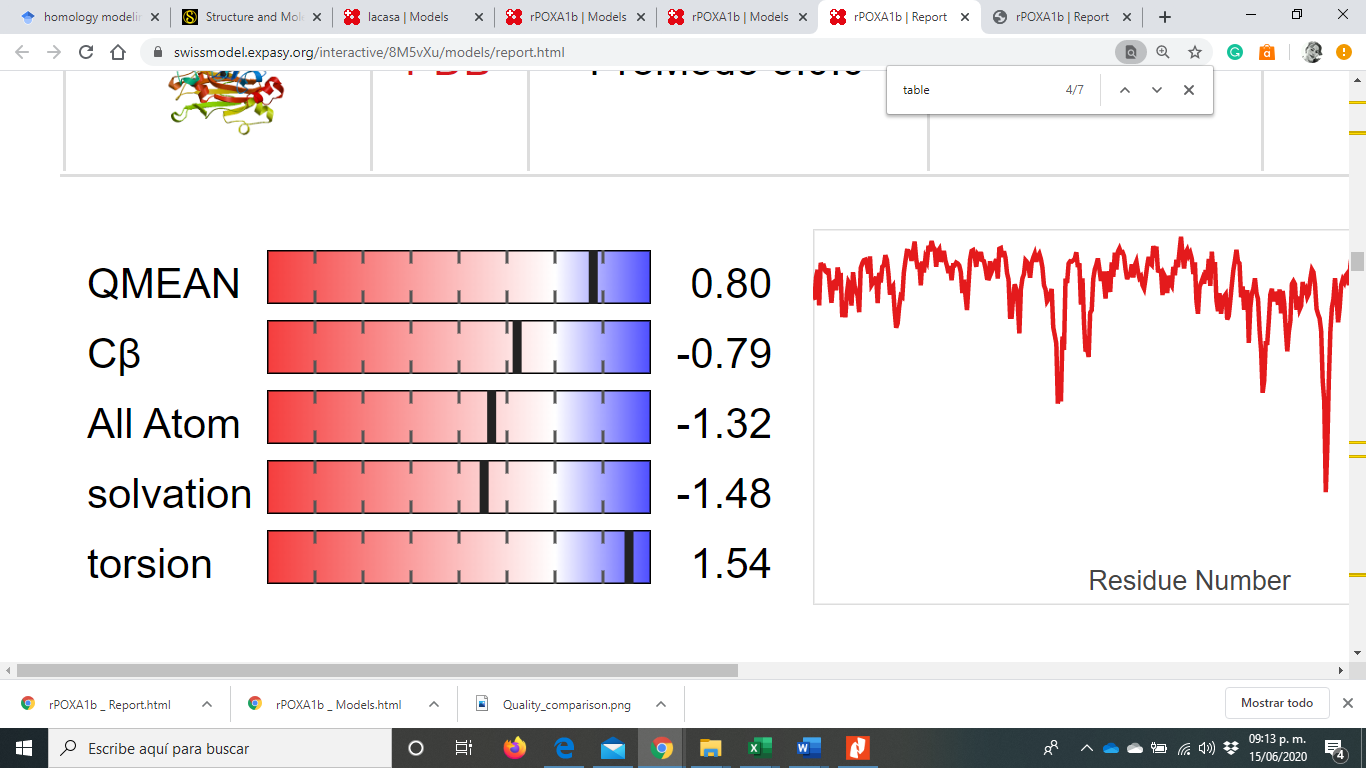


A

B

Supplementary Material Fig. 1 QMEAN Scores A) Global model quality estimation. B) Comparison with a non-redundant set of PDB structures.


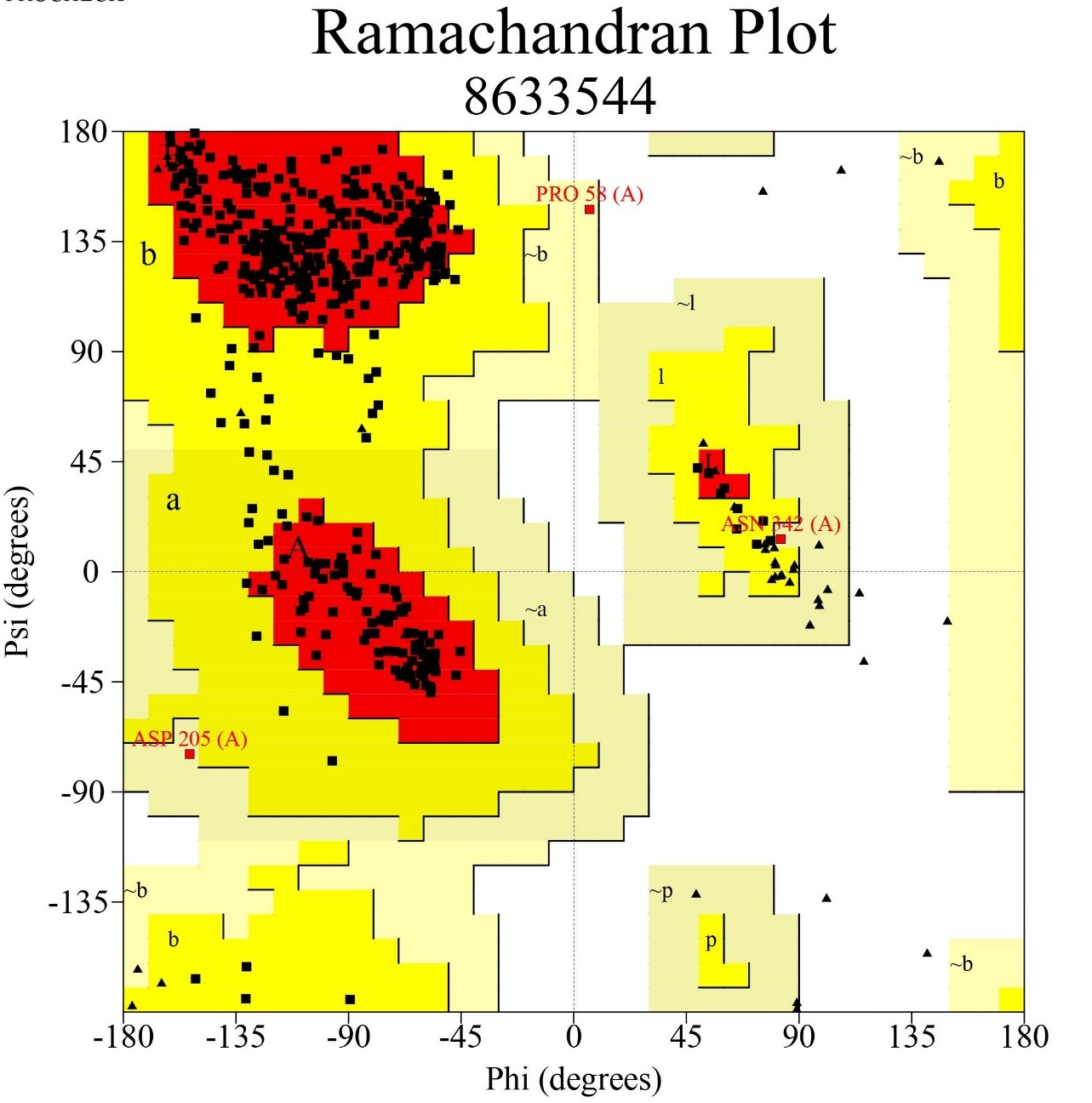


| **Plot statistics** |  |  |
| --- | --- | --- |
| Residues in most favoured regions [A,B,L] | 360 | 88.9% |
| Residues in additional allowed regions [a,b,l,p] | 43 | 10.6% |
| Residues in generously allowed regions [~a,~b,~l,~p] | 2 | 0.5% |
| Residues in disallowed regions | 0 | 0.0% |

Supplementary Material Fig. 2. Ramachandran plot of laccase 3D model of POXA 1B from *P. ostreatus*


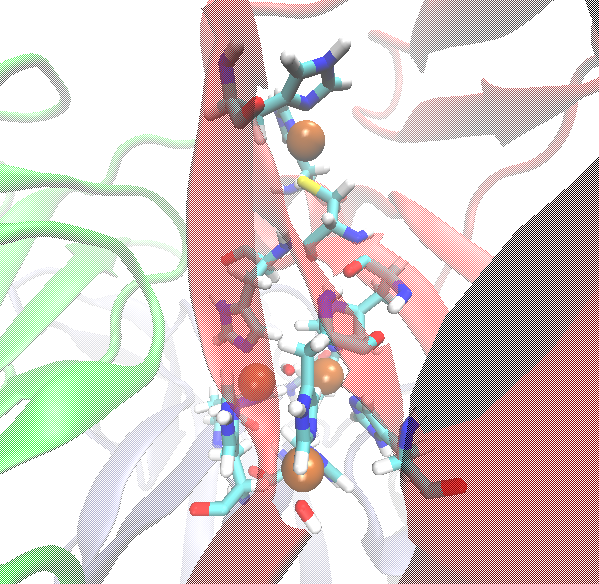


Supplementary Material Fig. 3. Structural representation of copper atom coordination forming POXA 1B active site.


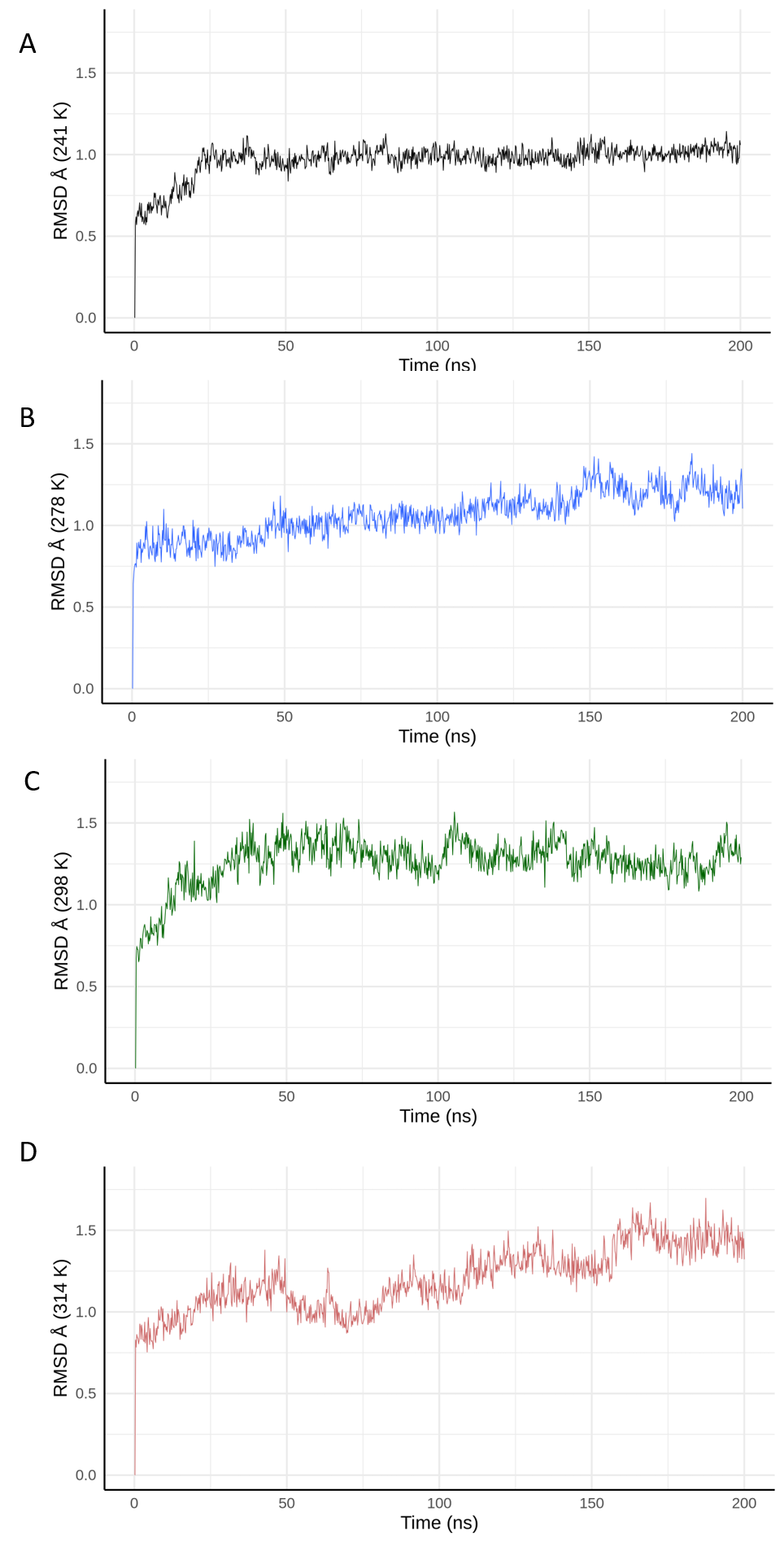


Supplementary Material Fig. 4. Root Mean Square Deviation (RMSD) as a function of time during the MD simulations of POXA 1B. **A**. 241 K (-32.15 °C), **B**. 278 K (4.85 °C), **C**. 298 K (24.85 °C) and **D**. 314 K (40.85 °C).


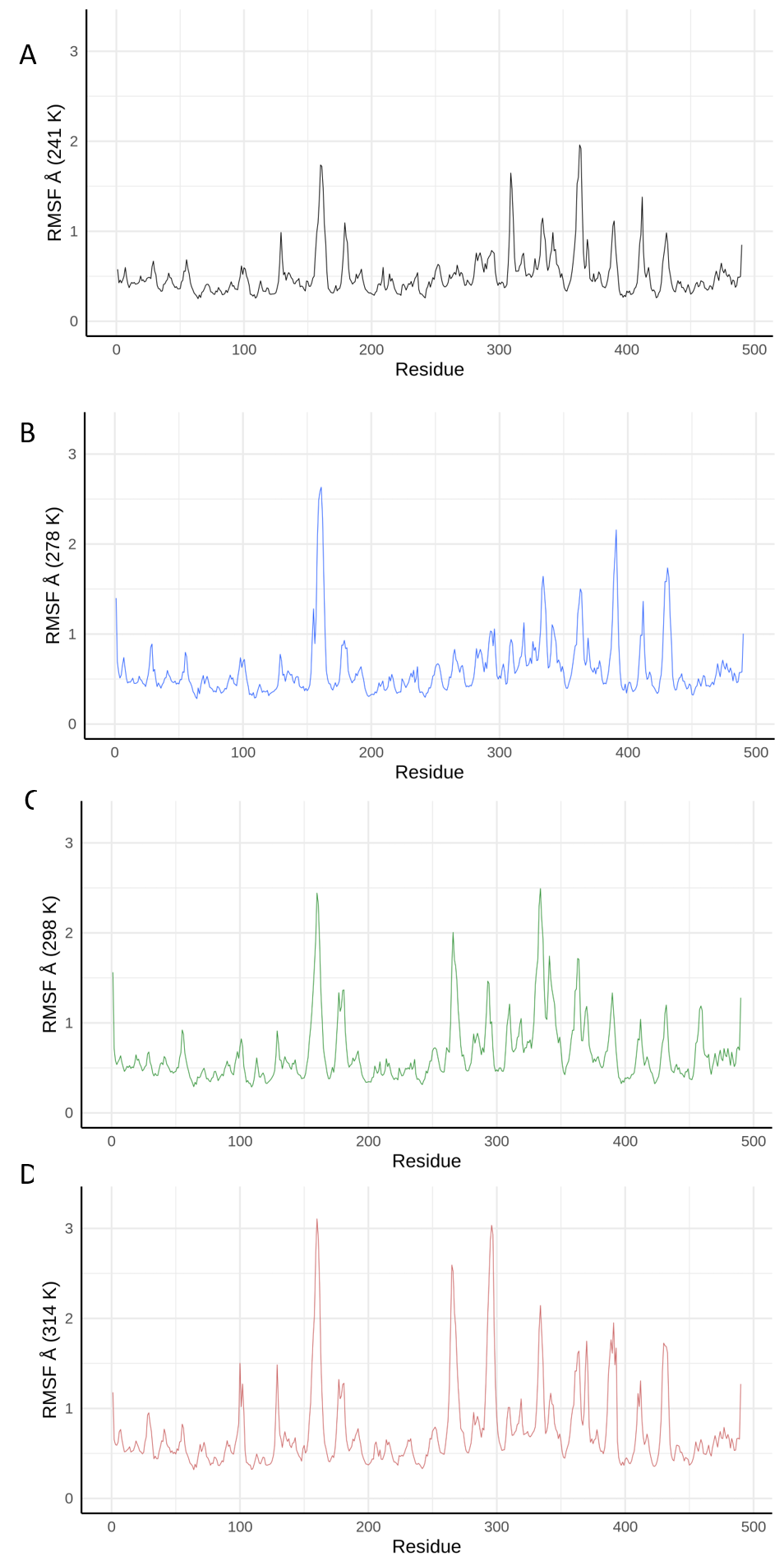


Supplementary Material Fig. 5 Root mean square ﬂuctuation (Å) analyses at different temperatures. A. 214 K, B. 278 K, C. 298 K, D. 314 K.


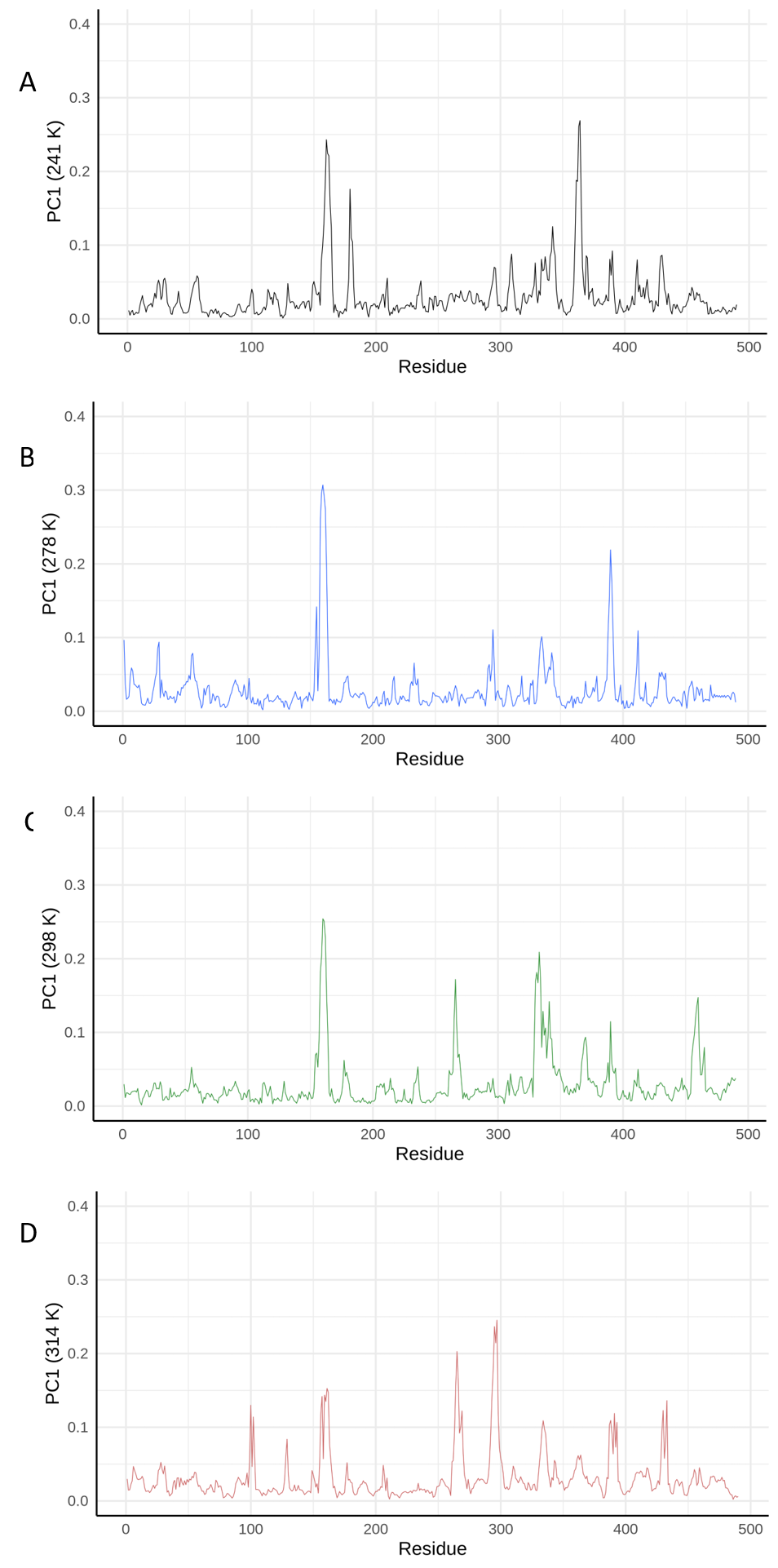


Supplementary Material Fig. 6 PC1 collective movements for all evaluated temperatures. A. 214 K, B. 278 K, C. 298 K, D. 314 K

A


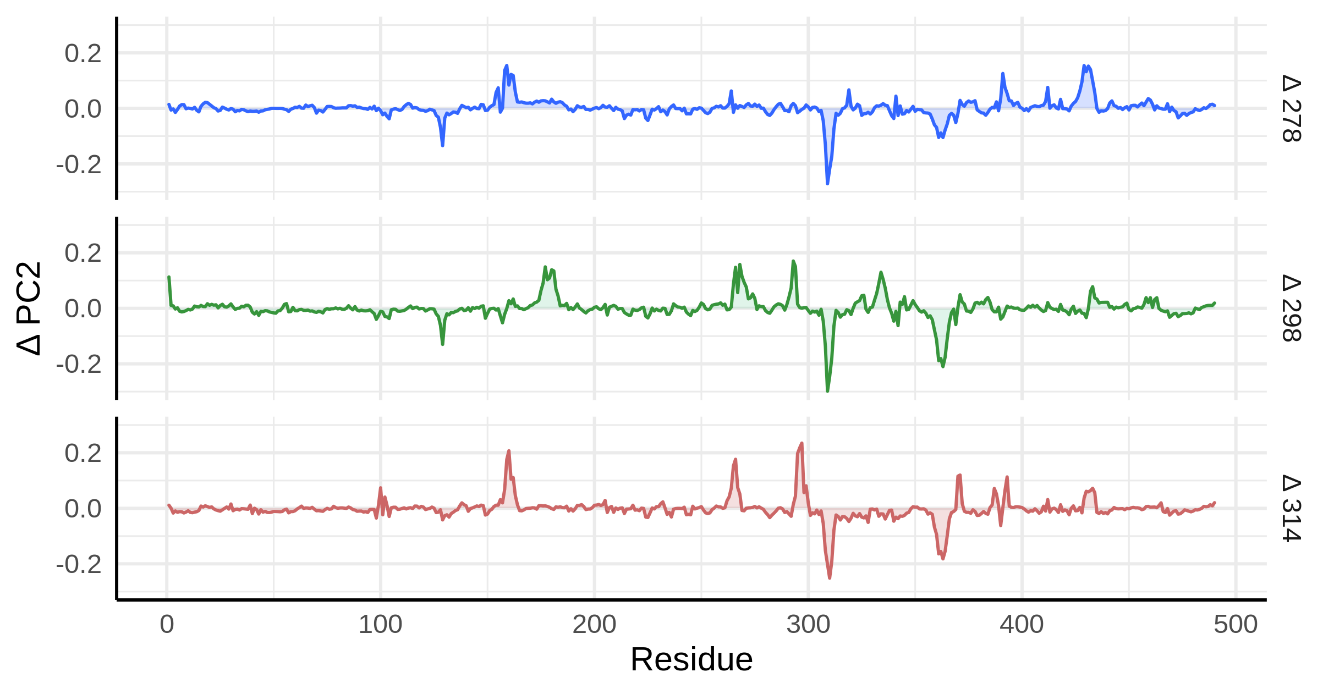


B


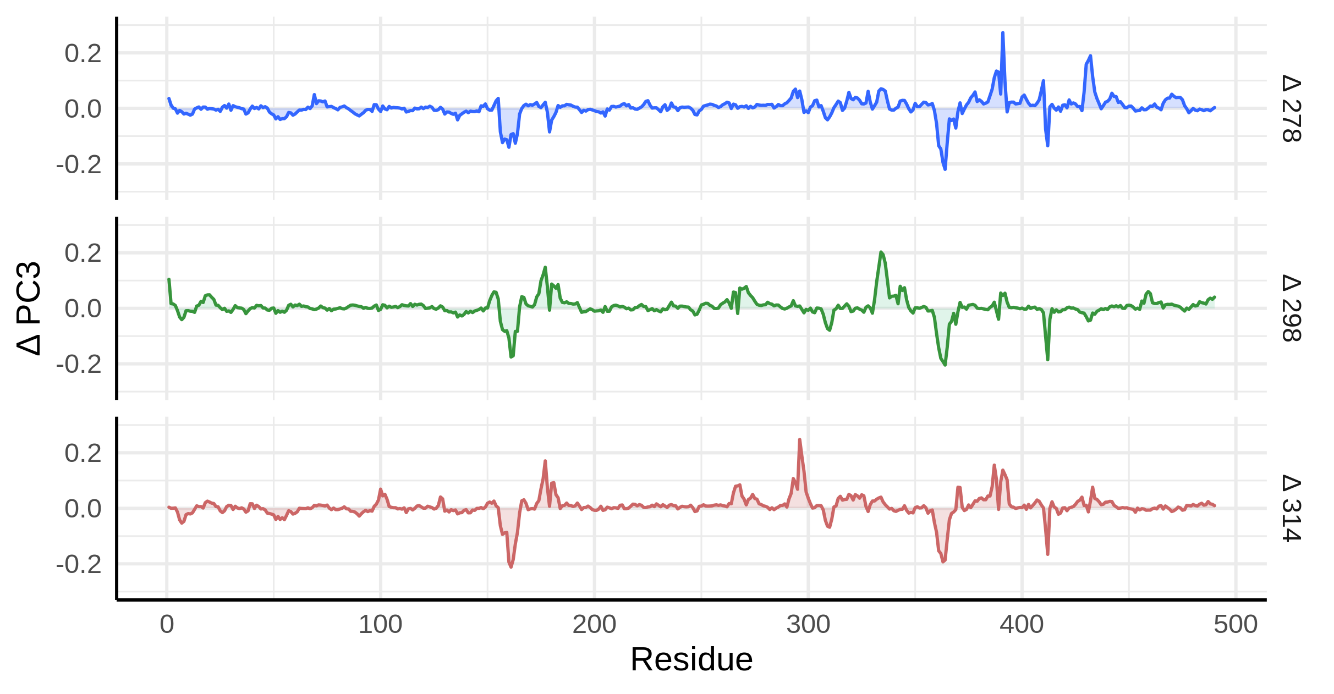


Supplementary Material Fig. 7. ∆ PC calculated from the difference of the PCA average of the simulations between different temperatures: 278, 298 and 314 K with 241 K (278-241 K red; 278-241 K green and 314-241 K blue. **A.** ∆ PC2 and **B.** ∆ PC3


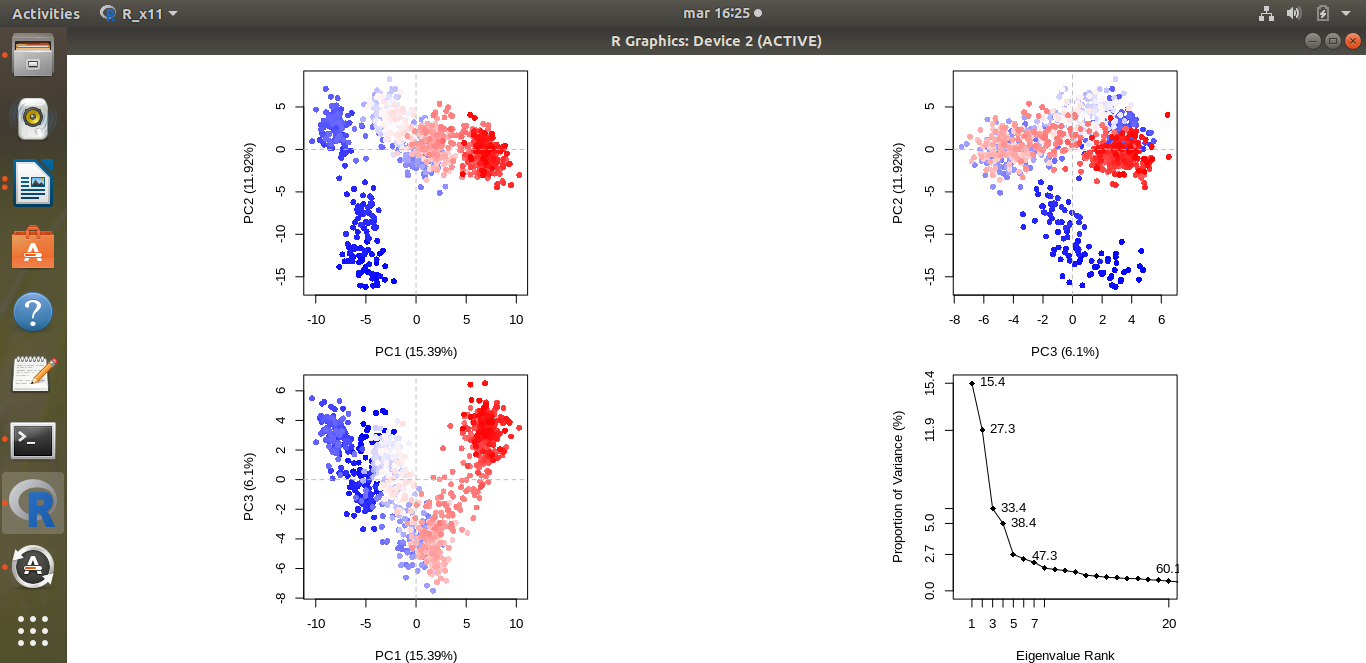

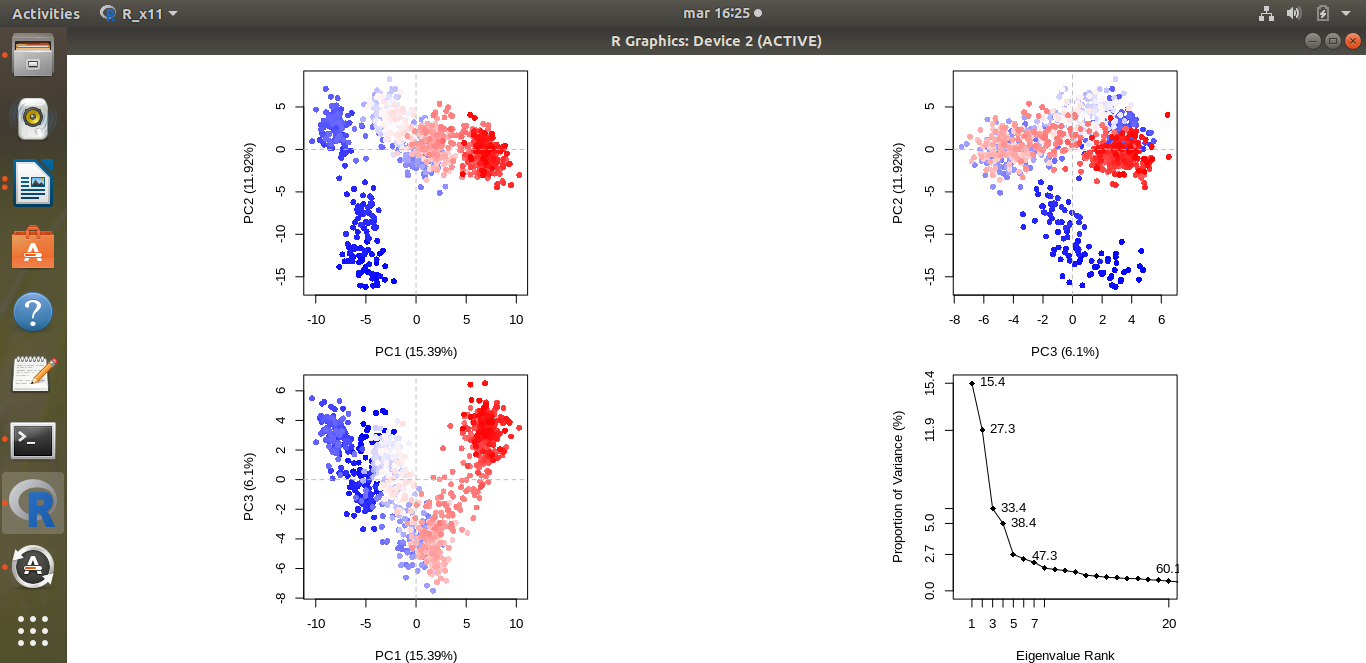

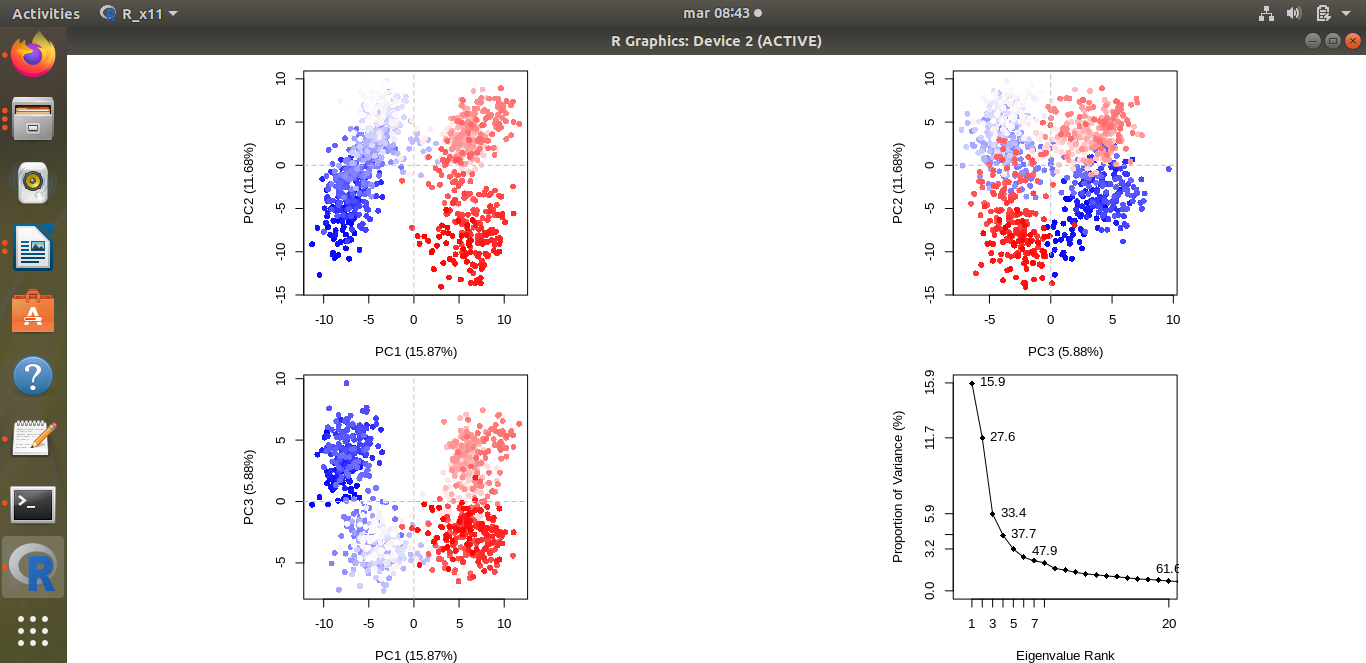

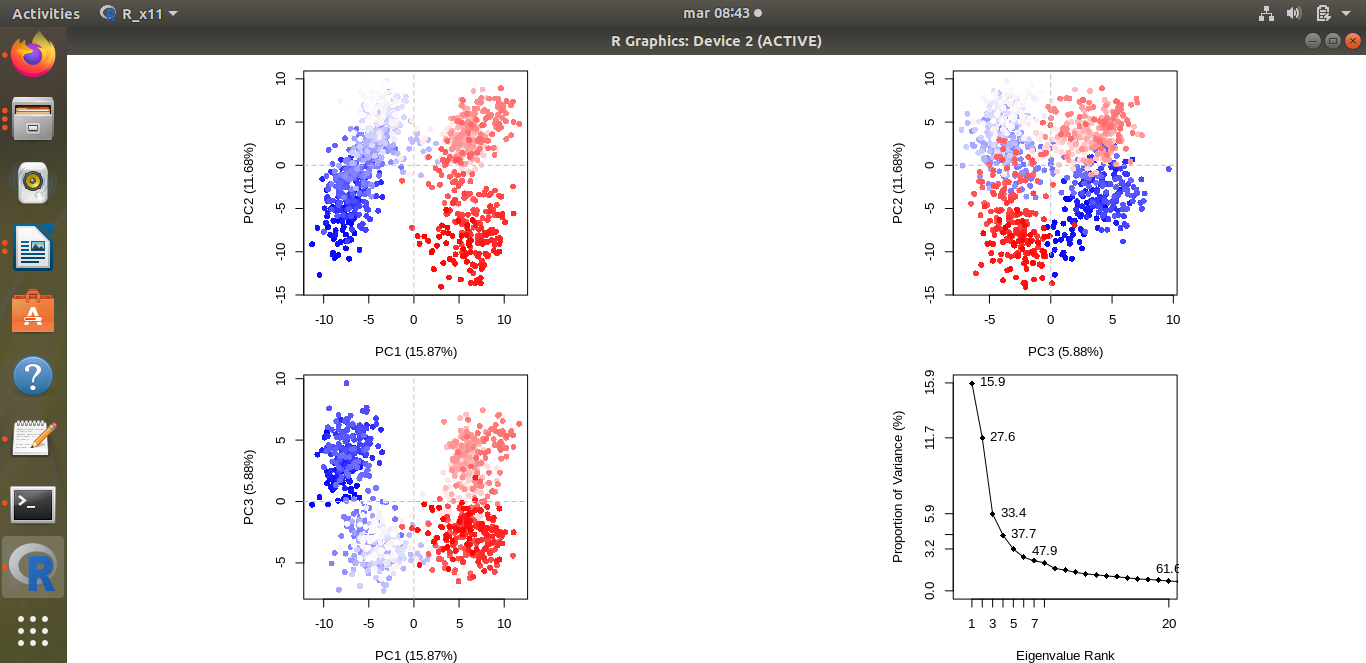


A

B


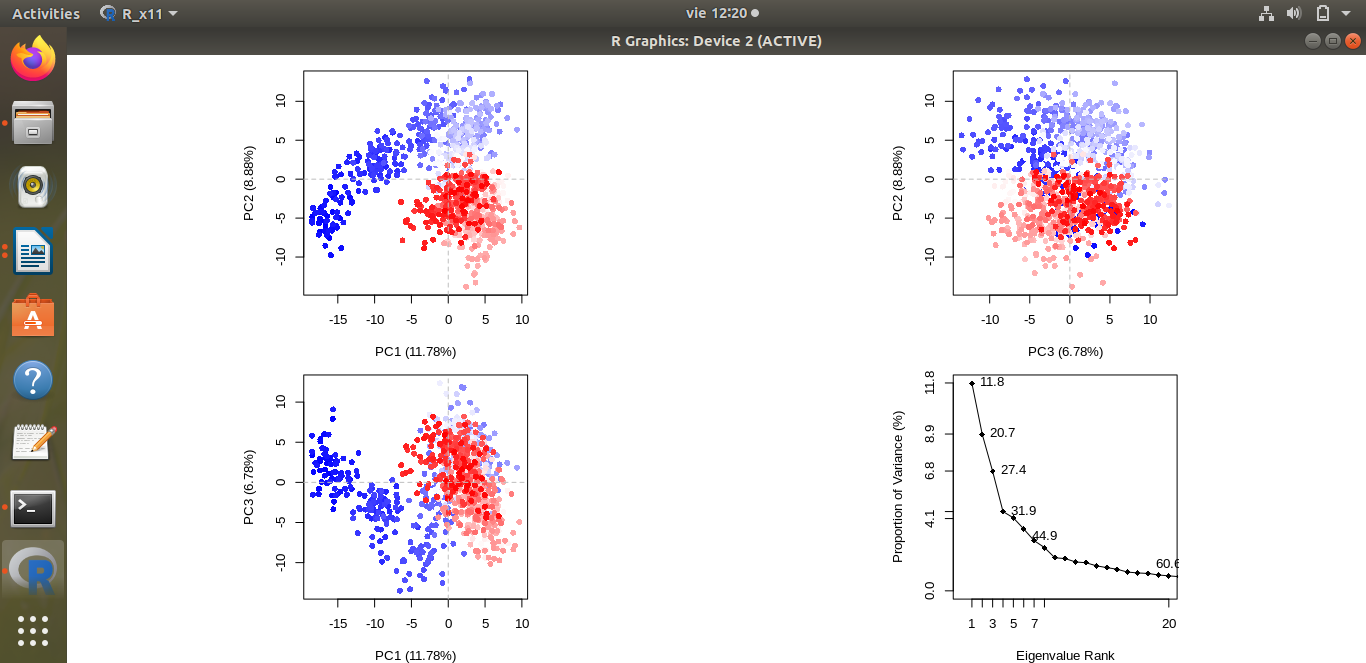

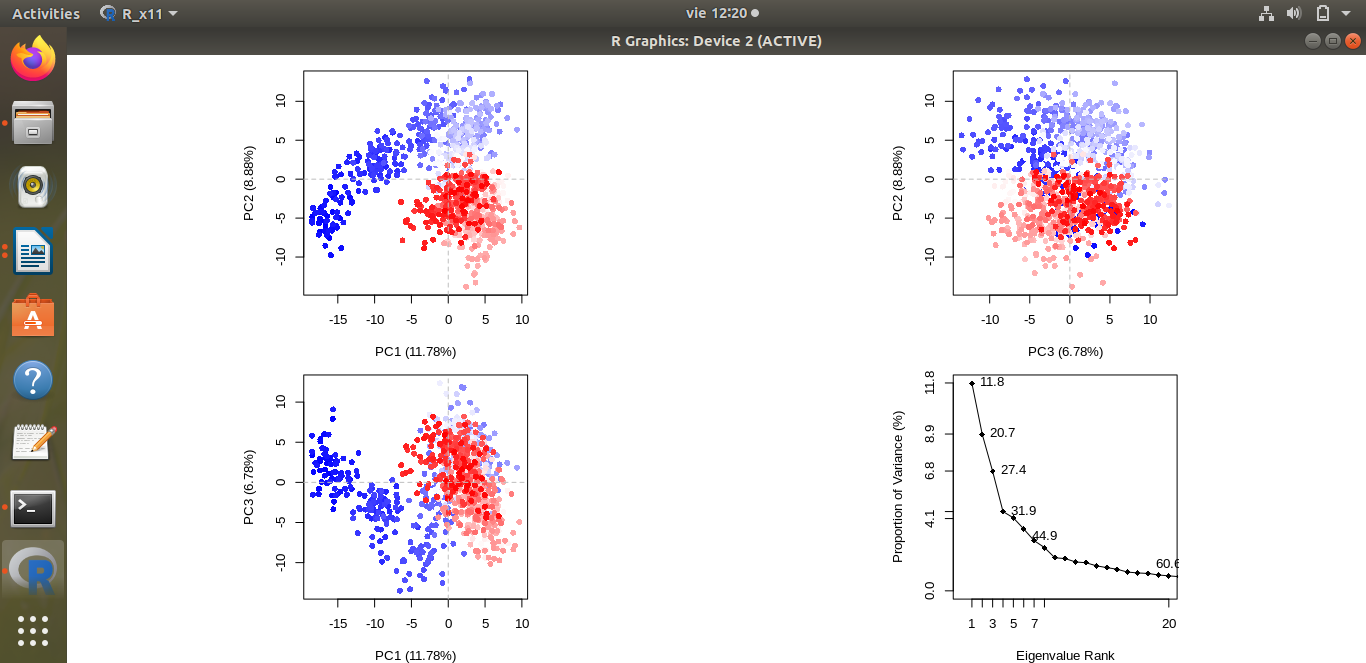


C

D


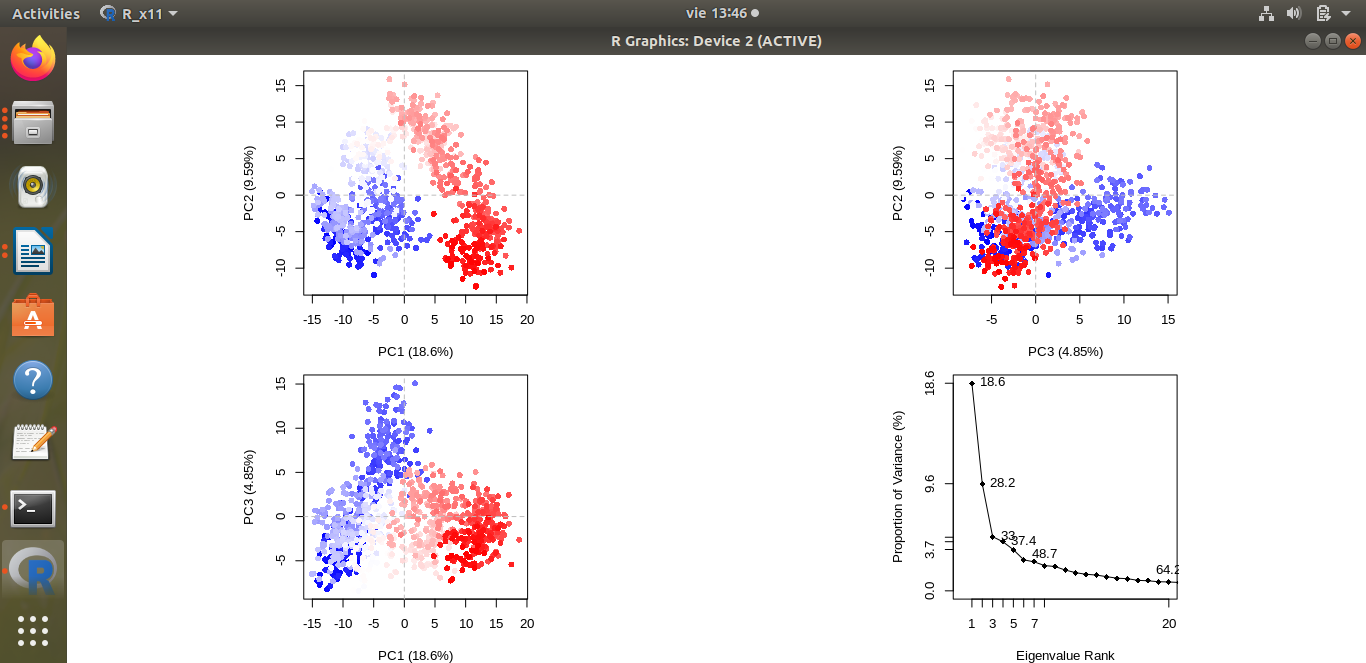

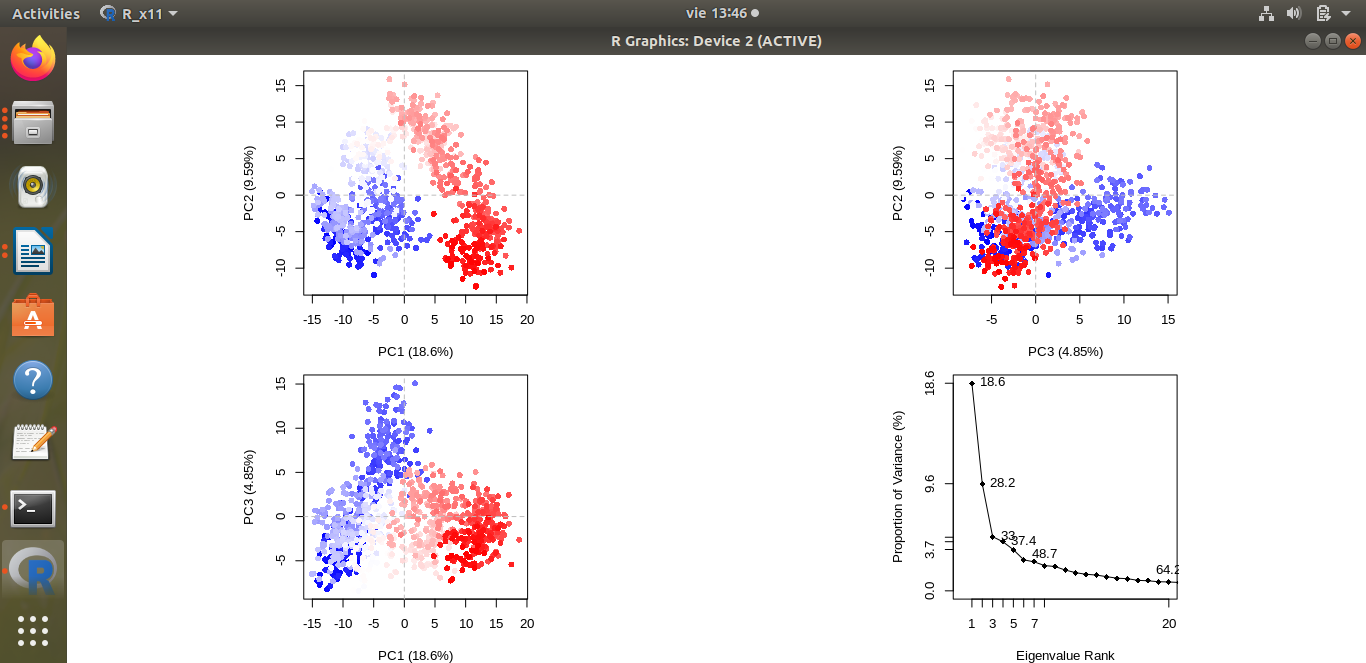


Supplementary Material Fig. 8. POXA 1B Principal Component Analysis (PCA) at different temperatures. **A**. 241 K (-32.15 °C), **B**. 278 K (4.85 °C), **C**. 298 K (24.85 °C) and **D**. 314 K (40.85 °C).

Supplementary Materia Sl. File generated by mcpb.py with force field parameters.

MASS

M1 63.55 Cu ion

M2 63.55 Cu ion

M3 63.55 Cu ion

M4 63.55 Cu ion

Y1 14.01 0.530 sp2 N in 5 memb.ring w/LP (HIS,ADE,GUA)

Y2 14.01 0.530 sp2 N in 5 memb.ring w/LP (HIS,ADE,GUA)

Y5 14.01 0.530 sp2 N in 5 memb.ring w/LP (HIS,ADE,GUA)

Y6 14.01 0.530 sp2 N in 5 memb.ring w/LP (HIS,ADE,GUA)

Y7 14.01 0.530 sp2 N in 5 memb.ring w/LP (HIS,ADE,GUA)

Y3 14.01 0.530 sp2 N in 5 memb.ring w/LP (HIS,ADE,GUA)

Y8 14.01 0.530 sp2 N in 5 memb.ring w/LP (HIS,ADE,GUA)

Y9 14.01 0.530 sp2 N in 5 memb.ring w/LP (HIS,ADE,GUA)

Z1 14.01 0.530 sp2 N in 5 memb.ring w/LP (HIS,ADE,GUA)

Z2 32.06 2.900 S in cystine

Z3 14.01 0.530 sp2 N in 5 memb.ring w/LP (HIS,ADE,GUA)

Z4 16.00 0.000 oxygen in TIP3P water

Y4 16.00 0.000 oxygen in TIP3P water

BOND

M1-Y4 73.6 1.9223 Created by Seminario method using MCPB.py

M2-Y4 70.8 1.9303 Created by Seminario method using MCPB.py

Y1-M1 80.9 1.9892 Created by Seminario method using MCPB.py

Y2-M1 89.1 1.9526 Created by Seminario method using MCPB.py

Y3-M1 88.0 1.9567 Created by Seminario method using MCPB.py

Y5-M2 72.6 1.9851 Created by Seminario method using MCPB.py

Y6-M2 81.7 1.9781 Created by Seminario method using MCPB.py

Y7-M2 79.2 1.9662 Created by Seminario method using MCPB.py

CC-Y1 410.0 1.394 JCC,7,(1986),230; HIS

CR-Y2 488.0 1.335 JCC,7,(1986),230; HIS

CR-Y3 488.0 1.335 JCC,7,(1986),230; HIS

CR-Y5 488.0 1.335 JCC,7,(1986),230; HIS

CR-Y6 488.0 1.335 JCC,7,(1986),230; HIS

CR-Y7 488.0 1.335 JCC,7,(1986),230; HIS

Y1-CR 488.0 1.335 JCC,7,(1986),230; HIS

Y2-CV 410.0 1.394 JCC,7,(1986),230; HIS

Y3-CV 410.0 1.394 JCC,7,(1986),230; HIS

Y4-HW 553.0 0.9572 TIP3P water

Y5-CV 410.0 1.394 JCC,7,(1986),230; HIS

Y6-CV 410.0 1.394 JCC,7,(1986),230; HIS

Y7-CV 410.0 1.394 JCC,7,(1986),230; HIS

M3-Z4 250.6 1.7554 Created by Seminario method using MCPB.py

Y8-M3 113.6 1.9241 Created by Seminario method using MCPB.py

Y9-M3 111.7 1.9235 Created by Seminario method using MCPB.py

CR-Y8 488.0 1.335 JCC,7,(1986),230; HIS

CR-Y9 488.0 1.335 JCC,7,(1986),230; HIS

Y8-CV 410.0 1.394 JCC,7,(1986),230; HIS

Y9-CV 410.0 1.394 JCC,7,(1986),230; HIS

Z4-HW 553.0 0.9572 TIP3P water

Z1-M4 96.9 1.9464 Created by Seminario method using MCPB.py

Z2-M4 122.8 2.1263 Created by Seminario method using MCPB.py

Z3-M4 98.5 1.9411 Created by Seminario method using MCPB.py

CC-Z1 410.0 1.394 JCC,7,(1986),230; HIS

CC-Z3 410.0 1.394 JCC,7,(1986),230; HIS

CT-Z2 237.0 1.810 changed from 222.0 based on methanethiol nmodes

Z1-CR 488.0 1.335 JCC,7,(1986),230; HIS

Z3-CR 488.0 1.335 JCC,7,(1986),230; HIS

ANGL

CC-Y1-M1 111.34 127.17 Created by Seminario method using MCPB.py

CR-Y2-M1 94.44 128.31 Created by Seminario method using MCPB.py

CR-Y3-M1 81.82 126.20 Created by Seminario method using MCPB.py

CR-Y5-M2 69.32 124.29 Created by Seminario method using MCPB.py

CR-Y6-M2 94.04 127.85 Created by Seminario method using MCPB.py

CR-Y7-M2 73.79 126.75 Created by Seminario method using MCPB.py

M1-Y1-CR 105.52 125.29 Created by Seminario method using MCPB.py

M1-Y2-CV 99.76 125.07 Created by Seminario method using MCPB.py

M1-Y3-CV 83.13 126.97 Created by Seminario method using MCPB.py

M1-Y4-HW 28.61 100.53 Created by Seminario method using MCPB.py

M1-Y4-M2 74.12 142.03 Created by Seminario method using MCPB.py

M2-Y4-HW 24.20 104.59 Created by Seminario method using MCPB.py

M2-Y5-CV 78.15 128.87 Created by Seminario method using MCPB.py

M2-Y6-CV 96.05 126.00 Created by Seminario method using MCPB.py

M2-Y7-CV 77.79 125.53 Created by Seminario method using MCPB.py

Y1-M1-Y2 57.10 94.40 Created by Seminario method using MCPB.py

Y1-M1-Y3 60.64 95.55 Created by Seminario method using MCPB.py

Y1-M1-Y4 58.22 154.73 Created by Seminario method using MCPB.py

Y2-M1-Y3 48.44 149.23 Created by Seminario method using MCPB.py

Y2-M1-Y4 77.39 89.33 Created by Seminario method using MCPB.py

Y3-M1-Y4 86.84 93.86 Created by Seminario method using MCPB.py

Y5-M2-Y4 63.50 92.72 Created by Seminario method using MCPB.py

Y5-M2-Y6 43.14 98.05 Created by Seminario method using MCPB.py

Y5-M2-Y7 36.57 137.23 Created by Seminario method using MCPB.py

Y6-M2-Y4 64.16 145.17 Created by Seminario method using MCPB.py

Y6-M2-Y7 54.39 99.45 Created by Seminario method using MCPB.py

Y7-M2-Y4 61.10 94.63 Created by Seminario method using MCPB.py

CC-CV-Y2 70.0 120.00 AA his

CC-CV-Y3 70.0 120.00 AA his

CC-CV-Y5 70.0 120.00 AA his

CC-CV-Y6 70.0 120.00 AA his

CC-CV-Y7 70.0 120.00 AA his

CC-Y1-CR 70.0 117.00 AA his

CR-Y2-CV 70.0 117.00 AA his

CR-Y3-CV 70.0 117.00 AA his

CR-Y5-CV 70.0 117.00 AA his

CR-Y6-CV 70.0 117.00 AA his

CR-Y7-CV 70.0 117.00 AA his

CT-CC-Y1 70.0 120.00 AA his

CW-CC-Y1 70.0 120.00 AA his

NA-CR-Y2 70.0 120.00 AA his

NA-CR-Y3 70.0 120.00 AA his

NA-CR-Y5 70.0 120.00 AA his

NA-CR-Y6 70.0 120.00 AA his

NA-CR-Y7 70.0 120.00 AA his

Y1-CR-H5 50.0 120.00 AA his

Y1-CR-NA 70.0 120.00 AA his

Y2-CR-H5 50.0 120.00 AA his

Y2-CV-H4 50.0 120.00 AA his

Y3-CR-H5 50.0 120.00 AA his

Y3-CV-H4 50.0 120.00 AA his

Y5-CR-H5 50.0 120.00 AA his

Y5-CV-H4 50.0 120.00 AA his

Y6-CR-H5 50.0 120.00 AA his

Y6-CV-H4 50.0 120.00 AA his

Y7-CR-H5 50.0 120.00 AA his

Y7-CV-H4 50.0 120.00 AA his

CR-Y8-M3 73.02 126.02 Created by Seminario method using MCPB.py

CR-Y9-M3 61.34 121.54 Created by Seminario method using MCPB.py

M3-Y8-CV 74.77 127.14 Created by Seminario method using MCPB.py

M3-Y9-CV 65.58 131.64 Created by Seminario method using MCPB.py

M3-Z4-HW 17.92 122.05 Created by Seminario method using MCPB.py

Y8-M3-Y9 67.95 103.13 Created by Seminario method using MCPB.py

Y8-M3-Z4 50.17 140.40 Created by Seminario method using MCPB.py

Y9-M3-Z4 34.59 115.41 Created by Seminario method using MCPB.py

CC-CV-Y8 70.0 120.00 AA his

CC-CV-Y9 70.0 120.00 AA his

CR-Y8-CV 70.0 117.00 AA his

CR-Y9-CV 70.0 117.00 AA his

NA-CR-Y8 70.0 120.00 AA his

NA-CR-Y9 70.0 120.00 AA his

Y8-CR-H5 50.0 120.00 AA his

Y8-CV-H4 50.0 120.00 AA his

Y9-CR-H5 50.0 120.00 AA his

Y9-CV-H4 50.0 120.00 AA his

CC-Z1-M4 68.11 125.52 Created by Seminario method using MCPB.py

CC-Z3-M4 69.77 128.37 Created by Seminario method using MCPB.py

CT-Z2-M4 87.22 108.96 Created by Seminario method using MCPB.py

M4-Z1-CR 70.51 126.79 Created by Seminario method using MCPB.py

M4-Z3-CR 73.98 124.05 Created by Seminario method using MCPB.py

Z1-M4-Z2 19.83 128.11 Created by Seminario method using MCPB.py

Z1-M4-Z3 61.67 101.44 Created by Seminario method using MCPB.py

Z2-M4-Z3 28.31 129.95 Created by Seminario method using MCPB.py

CC-Z1-CR 70.0 117.00 AA his

CC-Z3-CR 70.0 117.00 AA his

CT-CC-Z1 70.0 120.00 AA his

CT-CC-Z3 70.0 120.00 AA his

CW-CC-Z1 70.0 120.00 AA his

CW-CC-Z3 70.0 120.00 AA his

CX-CT-Z2 50.0 108.60 AA cys (was CT-CT-SH)

Z1-CR-H5 50.0 120.00 AA his

Z1-CR-NA 70.0 120.00 AA his

Z2-CT-H1 50.0 109.50 AA cyx changed based on NMA nmodes

Z3-CR-H5 50.0 120.00 AA his

Z3-CR-NA 70.0 120.00 AA his

CC-Z1-M4 68.11 125.52 Created by Seminario method using MCPB.py

CC-Z3-M4 69.77 128.37 Created by Seminario method using MCPB.py

CT-Z2-M4 87.22 108.96 Created by Seminario method using MCPB.py

M4-Z1-CR 70.51 126.79 Created by Seminario method using MCPB.py

M4-Z3-CR 73.98 124.05 Created by Seminario method using MCPB.py

Z1-M4-Z2 19.83 128.11 Created by Seminario method using MCPB.py

Z1-M4-Z3 61.67 101.44 Created by Seminario method using MCPB.py

Z2-M4-Z3 28.31 129.95 Created by Seminario method using MCPB.py

CC-Z1-CR 70.0 117.00 AA his

CC-Z3-CR 70.0 117.00 AA his

CT-CC-Z1 70.0 120.00 AA his

CT-CC-Z3 70.0 120.00 AA his

CW-CC-Z1 70.0 120.00 AA his

CW-CC-Z3 70.0 120.00 AA his

CX-CT-Z2 50.0 108.60 AA cys (was CT-CT-SH)

Z1-CR-H5 50.0 120.00 AA his

Z1-CR-NA 70.0 120.00 AA his

Z2-CT-H1 50.0 109.50 AA cyx changed based on NMA nmodes

Z3-CR-H5 50.0 120.00 AA his

Z3-CR-NA 70.0 120.00 AA his

DIHE

X -CC-Y1-X 2 4.8 180.0 2.0 JCC,7,(1986),230

X -CR-Y2-X 2 10.0 180.0 2.0 JCC,7,(1986),230

X -CR-Y3-X 2 10.0 180.0 2.0 JCC,7,(1986),230

X -CR-Y5-X 2 10.0 180.0 2.0 JCC,7,(1986),230

X -CR-Y6-X 2 10.0 180.0 2.0 JCC,7,(1986),230

X -CR-Y7-X 2 10.0 180.0 2.0 JCC,7,(1986),230

X -CV-Y2-X 2 4.8 180.0 2.0 JCC,7,(1986),230

X -CV-Y3-X 2 4.8 180.0 2.0 JCC,7,(1986),230

X -CV-Y5-X 2 4.8 180.0 2.0 JCC,7,(1986),230

X -CV-Y6-X 2 4.8 180.0 2.0 JCC,7,(1986),230

X -CV-Y7-X 2 4.8 180.0 2.0 JCC,7,(1986),230

X -Y1-CR-X 2 10.0 180.0 2.0 JCC,7,(1986),230

CC-CV-Y2-M1 3 0.00 0.00 3.0 Treat as zero by MCPB.py

CC-CV-Y3-M1 3 0.00 0.00 3.0 Treat as zero by MCPB.py

CC-CV-Y5-M2 3 0.00 0.00 3.0 Treat as zero by MCPB.py

CC-CV-Y6-M2 3 0.00 0.00 3.0 Treat as zero by MCPB.py

CC-CV-Y7-M2 3 0.00 0.00 3.0 Treat as zero by MCPB.py

CC-Y1-M1-Y2 3 0.00 0.00 3.0 Treat as zero by MCPB.py

CC-Y1-M1-Y3 3 0.00 0.00 3.0 Treat as zero by MCPB.py

CC-Y1-M1-Y4 3 0.00 0.00 3.0 Treat as zero by MCPB.py

CR-Y2-M1-Y3 3 0.00 0.00 3.0 Treat as zero by MCPB.py

CR-Y2-M1-Y4 3 0.00 0.00 3.0 Treat as zero by MCPB.py

CR-Y3-M1-Y4 3 0.00 0.00 3.0 Treat as zero by MCPB.py

CR-Y5-M2-Y4 3 0.00 0.00 3.0 Treat as zero by MCPB.py

CR-Y5-M2-Y6 3 0.00 0.00 3.0 Treat as zero by MCPB.py

CR-Y5-M2-Y7 3 0.00 0.00 3.0 Treat as zero by MCPB.py

CR-Y6-M2-Y4 3 0.00 0.00 3.0 Treat as zero by MCPB.py

CR-Y6-M2-Y7 3 0.00 0.00 3.0 Treat as zero by MCPB.py

CR-Y7-M2-Y4 3 0.00 0.00 3.0 Treat as zero by MCPB.py

CT-CC-Y1-M1 3 0.00 0.00 3.0 Treat as zero by MCPB.py

CW-CC-Y1-M1 3 0.00 0.00 3.0 Treat as zero by MCPB.py

CX-CT-CC-Y1 1 0.047 180.0 -4.0

CX-CT-CC-Y1 1 0.74 0.0 -3.0

CX-CT-CC-Y1 1 0.204 0.0 -2.0

CX-CT-CC-Y1 1 0.69 0.0 1.0

M1-Y1-CR-H5 3 0.00 0.00 3.0 Treat as zero by MCPB.py

M1-Y1-CR-NA 3 0.00 0.00 3.0 Treat as zero by MCPB.py

M1-Y2-CR-H5 3 0.00 0.00 3.0 Treat as zero by MCPB.py

M1-Y2-CV-H4 3 0.00 0.00 3.0 Treat as zero by MCPB.py

M1-Y3-CR-H5 3 0.00 0.00 3.0 Treat as zero by MCPB.py

M1-Y3-CV-H4 3 0.00 0.00 3.0 Treat as zero by MCPB.py

M2-Y5-CR-H5 3 0.00 0.00 3.0 Treat as zero by MCPB.py

M2-Y5-CV-H4 3 0.00 0.00 3.0 Treat as zero by MCPB.py

M2-Y6-CR-H5 3 0.00 0.00 3.0 Treat as zero by MCPB.py

M2-Y6-CV-H4 3 0.00 0.00 3.0 Treat as zero by MCPB.py

M2-Y7-CR-H5 3 0.00 0.00 3.0 Treat as zero by MCPB.py

M2-Y7-CV-H4 3 0.00 0.00 3.0 Treat as zero by MCPB.py

NA-CR-Y2-M1 3 0.00 0.00 3.0 Treat as zero by MCPB.py

NA-CR-Y3-M1 3 0.00 0.00 3.0 Treat as zero by MCPB.py

NA-CR-Y5-M2 3 0.00 0.00 3.0 Treat as zero by MCPB.py

NA-CR-Y6-M2 3 0.00 0.00 3.0 Treat as zero by MCPB.py

NA-CR-Y7-M2 3 0.00 0.00 3.0 Treat as zero by MCPB.py

Y1-M1-Y2-CR 3 0.00 0.00 3.0 Treat as zero by MCPB.py

Y1-M1-Y2-CV 3 0.00 0.00 3.0 Treat as zero by MCPB.py

Y1-M1-Y3-CR 3 0.00 0.00 3.0 Treat as zero by MCPB.py

Y1-M1-Y3-CV 3 0.00 0.00 3.0 Treat as zero by MCPB.py

Y1-M1-Y4-HW 3 0.00 0.00 3.0 Treat as zero by MCPB.py

Y1-M1-Y4-M2 3 0.00 0.00 3.0 Treat as zero by MCPB.py

Y2-M1-Y1-CR 3 0.00 0.00 3.0 Treat as zero by MCPB.py

Y2-M1-Y3-CR 3 0.00 0.00 3.0 Treat as zero by MCPB.py

Y2-M1-Y3-CV 3 0.00 0.00 3.0 Treat as zero by MCPB.py

Y2-M1-Y4-HW 3 0.00 0.00 3.0 Treat as zero by MCPB.py

Y2-M1-Y4-M2 3 0.00 0.00 3.0 Treat as zero by MCPB.py

Y3-M1-Y1-CR 3 0.00 0.00 3.0 Treat as zero by MCPB.py

Y3-M1-Y2-CV 3 0.00 0.00 3.0 Treat as zero by MCPB.py

Y3-M1-Y4-HW 3 0.00 0.00 3.0 Treat as zero by MCPB.py

Y3-M1-Y4-M2 3 0.00 0.00 3.0 Treat as zero by MCPB.py

Y4-M1-Y1-CR 3 0.00 0.00 3.0 Treat as zero by MCPB.py

Y4-M1-Y2-CV 3 0.00 0.00 3.0 Treat as zero by MCPB.py

Y4-M1-Y3-CV 3 0.00 0.00 3.0 Treat as zero by MCPB.py

Y4-M2-Y5-CV 3 0.00 0.00 3.0 Treat as zero by MCPB.py

Y4-M2-Y6-CV 3 0.00 0.00 3.0 Treat as zero by MCPB.py

Y4-M2-Y7-CV 3 0.00 0.00 3.0 Treat as zero by MCPB.py

Y5-M2-Y4-HW 3 0.00 0.00 3.0 Treat as zero by MCPB.py

Y5-M2-Y4-M1 3 0.00 0.00 3.0 Treat as zero by MCPB.py

Y5-M2-Y6-CR 3 0.00 0.00 3.0 Treat as zero by MCPB.py

Y5-M2-Y6-CV 3 0.00 0.00 3.0 Treat as zero by MCPB.py

Y5-M2-Y7-CR 3 0.00 0.00 3.0 Treat as zero by MCPB.py

Y5-M2-Y7-CV 3 0.00 0.00 3.0 Treat as zero by MCPB.py

Y6-M2-Y4-HW 3 0.00 0.00 3.0 Treat as zero by MCPB.py

Y6-M2-Y4-M1 3 0.00 0.00 3.0 Treat as zero by MCPB.py

Y6-M2-Y5-CV 3 0.00 0.00 3.0 Treat as zero by MCPB.py

Y6-M2-Y7-CR 3 0.00 0.00 3.0 Treat as zero by MCPB.py

Y6-M2-Y7-CV 3 0.00 0.00 3.0 Treat as zero by MCPB.py

Y7-M2-Y4-HW 3 0.00 0.00 3.0 Treat as zero by MCPB.py

Y7-M2-Y4-M1 3 0.00 0.00 3.0 Treat as zero by MCPB.py

Y7-M2-Y5-CV 3 0.00 0.00 3.0 Treat as zero by MCPB.py

Y7-M2-Y6-CV 3 0.00 0.00 3.0 Treat as zero by MCPB.py

X -CR-Y8-X 2 10.0 180.0 2.0 JCC,7,(1986),230

X -CR-Y9-X 2 10.0 180.0 2.0 JCC,7,(1986),230

X -CV-Y8-X 2 4.8 180.0 2.0 JCC,7,(1986),230

X -CV-Y9-X 2 4.8 180.0 2.0 JCC,7,(1986),230

CC-CV-Y8-M3 3 0.00 0.00 3.0 Treat as zero by MCPB.py

CC-CV-Y9-M3 3 0.00 0.00 3.0 Treat as zero by MCPB.py

CR-Y8-M3-Y9 3 0.00 0.00 3.0 Treat as zero by MCPB.py

CR-Y8-M3-Z4 3 0.00 0.00 3.0 Treat as zero by MCPB.py

CR-Y9-M3-Z4 3 0.00 0.00 3.0 Treat as zero by MCPB.py

M3-Y8-CR-H5 3 0.00 0.00 3.0 Treat as zero by MCPB.py

M3-Y8-CV-H4 3 0.00 0.00 3.0 Treat as zero by MCPB.py

M3-Y9-CR-H5 3 0.00 0.00 3.0 Treat as zero by MCPB.py

M3-Y9-CV-H4 3 0.00 0.00 3.0 Treat as zero by MCPB.py

NA-CR-Y8-M3 3 0.00 0.00 3.0 Treat as zero by MCPB.py

NA-CR-Y9-M3 3 0.00 0.00 3.0 Treat as zero by MCPB.py

Y8-M3-Y9-CR 3 0.00 0.00 3.0 Treat as zero by MCPB.py

Y8-M3-Y9-CV 3 0.00 0.00 3.0 Treat as zero by MCPB.py

Y8-M3-Z4-HW 3 0.00 0.00 3.0 Treat as zero by MCPB.py

Y9-M3-Y8-CV 3 0.00 0.00 3.0 Treat as zero by MCPB.py

Y9-M3-Z4-HW 3 0.00 0.00 3.0 Treat as zero by MCPB.py

Z4-M3-Y8-CV 3 0.00 0.00 3.0 Treat as zero by MCPB.py

Z4-M3-Y9-CV 3 0.00 0.00 3.0 Treat as zero by MCPB.py

X -CC-Z1-X 2 4.8 180.0 2.0 JCC,7,(1986),230

X -CC-Z3-X 2 4.8 180.0 2.0 JCC,7,(1986),230

X -Z1-CR-X 2 10.0 180.0 2.0 JCC,7,(1986),230

X -Z3-CR-X 2 10.0 180.0 2.0 JCC,7,(1986),230

CC-Z1-M4-Z2 3 0.00 0.00 3.0 Treat as zero by MCPB.py

CC-Z1-M4-Z3 3 0.00 0.00 3.0 Treat as zero by MCPB.py

CT-CC-Z1-M4 3 0.00 0.00 3.0 Treat as zero by MCPB.py

CT-CC-Z3-M4 3 0.00 0.00 3.0 Treat as zero by MCPB.py

CT-Z2-M4-Z3 3 0.00 0.00 3.0 Treat as zero by MCPB.py

CW-CC-Z1-M4 3 0.00 0.00 3.0 Treat as zero by MCPB.py

CW-CC-Z3-M4 3 0.00 0.00 3.0 Treat as zero by MCPB.py

CX-CT-CC-Z1 1 0.047 180.0 -4.0

CX-CT-CC-Z1 1 0.74 0.0 -3.0

CX-CT-CC-Z1 1 0.204 0.0 -2.0

CX-CT-CC-Z1 1 0.69 0.0 1.0

CX-CT-CC-Z3 1 0.047 180.0 -4.0

CX-CT-CC-Z3 1 0.74 0.0 -3.0

CX-CT-CC-Z3 1 0.204 0.0 -2.0

CX-CT-CC-Z3 1 0.69 0.0 1.0

CX-CT-Z2-M4 3 0.00 0.00 3.0 Treat as zero by MCPB.py

M4-Z1-CR-H5 3 0.00 0.00 3.0 Treat as zero by MCPB.py

M4-Z1-CR-NA 3 0.00 0.00 3.0 Treat as zero by MCPB.py

M4-Z2-CT-H1 3 0.00 0.00 3.0 Treat as zero by MCPB.py

M4-Z3-CR-H5 3 0.00 0.00 3.0 Treat as zero by MCPB.py

M4-Z3-CR-NA 3 0.00 0.00 3.0 Treat as zero by MCPB.py

Z1-M4-Z2-CT 3 0.00 0.00 3.0 Treat as zero by MCPB.py

Z1-M4-Z3-CC 3 0.00 0.00 3.0 Treat as zero by MCPB.py

Z1-M4-Z3-CR 3 0.00 0.00 3.0 Treat as zero by MCPB.py

Z2-M4-Z1-CR 3 0.00 0.00 3.0 Treat as zero by MCPB.py

Z2-M4-Z3-CC 3 0.00 0.00 3.0 Treat as zero by MCPB.py

Z2-M4-Z3-CR 3 0.00 0.00 3.0 Treat as zero by MCPB.py

Z3-M4-Z1-CR 3 0.00 0.00 3.0 Treat as zero by MCPB.py

IMPR

CT-CW-CC-Y1 1.1 180. 2.

CT-CW-CC-Z3 1.1 180. 2.

CT-CW-CC-Z1 1.1 180. 2.

NONB

M1 1.4090 0.0172100000 IOD set for Cu2+ ion from Li et al. JCTC, 2013, 9, 2733

M2 1.4090 0.0172100000 IOD set for Cu2+ ion from Li et al. JCTC, 2013, 9, 2733

Y1 1.8240 0.1700 OPLS

Y2 1.8240 0.1700 OPLS

Y5 1.8240 0.1700 OPLS

Y6 1.8240 0.1700 OPLS

Y7 1.8240 0.1700 OPLS

Y3 1.8240 0.1700 OPLS

Y4 1.7683 0.1520 TIP3P water model

M3 1.4090 0.0172100000 IOD set for Cu2+ ion from Li et al. JCTC, 2013, 9, 2733

Y8 1.8240 0.1700 OPLS

Y9 1.8240 0.1700 OPLS

Z4 1.7683 0.1520 TIP3P water model

M4 1.4090 0.0172100000 IOD set for Cu2+ ion from Li et al. JCTC, 2013, 9, 2733

Z1 1.8240 0.1700 OPLS

Z2 2.0000 0.2500 W. Cornell CH3SH and CH3SCH3 FEP's

Z3 1.8240 0.1700 OPLS
